# Natural Corynanthe-Type Cholinesterase Inhibitors from Malaysian *Uncaria attenuata* Korth.: Isolation, Characterization, In Vitro and In Silico Studies

**DOI:** 10.3390/metabo13030390

**Published:** 2023-03-07

**Authors:** Nelson Jeng-Yeou Chear, Tan Ai Fein Ching-Ga, Kooi-Yeong Khaw, Francisco León, Wen-Nee Tan, Siti R. Yusof, Christopher R. McCurdy, Vikneswaran Murugaiyah, Surash Ramanathan

**Affiliations:** 1 Centre for Drug Research, Universiti Sains Malaysia, Minden 11800, Penang, Malaysia; 2 School of Pharmacy, Monash University Malaysia, Bandar Sunway 47500, Selangor, Malaysia; 3 Department of Drug Discovery and Biomedical Sciences, College of Pharmacy, University of South Carolina, Columbia, SC 29201, USA; 4 Chemistry Section, School of Distance Education, Universiti Sains Malaysia, Minden 11800, Penang, Malaysia; 5 Department of Medicinal Chemistry, College of Pharmacy, University of Florida, Gainesville, FL 32610, USA; 6 Discipline of Pharmacology, School of Pharmaceutical Sciences, Universiti Sains Malaysia, Minden 11800, Penang, Malaysia

**Keywords:** *Uncaria attenuata*, indole, oxindole, acetylcholinesterase, butyrylcholinesterase, molecular docking

## Abstract

The *Uncaria* genus is notable for its therapeutic potential in treating age-related dementia, such as Alzheimer’s disease. A phytochemical study of the leaves of Malaysian *Uncaria attenuata* Korth., afforded an undescribed natural corynanthe-type oxindole alkaloid, isovillocarine D (**1**) together with two known indole alkaloids, villocarine A (**2**) and geissoschizine methyl ether (**3**), and their structural identification was performed with extensive mono- and bidimensional NMR and MS spectroscopic methods. The isolated alkaloids were evaluated for their acetylcholinesterase (AChE)- and butyrylcholinesterase (BChE)-inhibitory activity. The results indicated that compound (**2**) was the most potent inhibitor against both AChE and BChE, with IC_50_ values of 14.45 and 13.95 µM, respectively, whereas compounds (**1**) and (**3**) were selective BChE inhibitors with IC_50_ values of 35.28 and 17.65 µM, respectively. In addition, molecular docking studies revealed that compound (**2**) interacts with the five main regions of AChE via both hydrogen and hydrophobic bonding. In contrast to AChE, the interactions of (**2**) with the enzymatic site of BChE are established only through hydrophobic bonding. The current finding suggests that *U. attenuata* could be a good source of bioactive alkaloids for treating age-related dementia.

## 1. Introduction

Alzheimer’s disease (AD) is the most prevalent type of age-related dementia among the elderly, which is characterized by a progressive decline in cognitive function and memory [1,2]. The classical hallmarks of AD pathogenesis are the formation of extracellular amyloid-β plaques, the accumulation of abnormally phosphorylated tau at the intracellular level, and the progressive loss of cholinergic neurons [3,4,5]. Unfortunately, most clinical trials targeting a single protein target, such as Aβ and tau, have often failed [6]. Current clinically approved anti-AD drugs are mainly cholinesterase inhibitors, such as rivastigmine, donepezil, and galantamine (Figure S1) [7,8]. Cholinesterase inhibitors block the action of cholinesterase, increasing acetylcholine availability and, subsequently, its duration of action in the brains of AD patients, which is essential for arousal, attention, learning, memory, muscle activation, etc. [9,10]. Nonetheless, cholinesterase inhibitors are symptomatic treatments that neither halt the disease course nor reverse the disease progression [11,12]. Due to the limited cholinesterase inhibitors available clinically, the search for more effective ones is ongoing, including from plant sources.

The *Uncaria* genus (Rubiaceae family), which includes *Uncaria rhynchopylla* (Gou-Teng), is commonly used in traditional Chinese medicine to treat cardiovascular and central nervous system disorders [2,13]. The main neuroprotective compounds in the *Uncaria* species are corynanthe-type indole and oxindole alkaloids such as rhynchophylline, isorhynchophylline, corynoxeine, isocorynoxeine, corynoxine, geissoschizine methyl ether, hirsuteine, and hirsutine (Figure S2a), which have been shown to have anti-amyloid aggregation, anti-tau hyperphosphorylation, anti-neuroinflammation, anti-aging, and cholinesterase-inhibitory activities, among others [13,14,15,16].

In our continuous effort to search for novel cholinesterase inhibitors from Malaysian Rubiaceous plants, the methanolic leaf extract of *Uncaria attenuata* Korth. has shown promising cholinesterase-inhibitory activity. *Uncaria attenuata* (also known as *Uncaria salaccinesis* or *Uncaria bulusanensis*) is a rare *Uncaria* species native to the Malay Archipelago (Peninsular Thailand, Malaysia, Indonesia, and the Philippines) [17,18]. A chemical investigation of *U. attenuata* was carried out between 1970 and 1997, of which the plant materials were collected from different localities in Thailand and Indonesia. Several common corynanthe- and heteroyohimbine-type alkaloids have been reported from the leaves of *U. attenuata*, including hirsuteine, hirsutine, rhynchophylline, isorhynchopylline, corynoxine B, mitraphylline, isomitraphylline, 3-isoajmalicine, tetrahydroalstonine, and rauniticine. However, the alkaloid profiles were greatly influenced by geographical origins [17,18,19]. In addition, four unusual _D_-secocorynanthe-type oxindole alkaloids—salacin, 3-oxo-7-hydroxy-3,7-secorhynchophylline, Us-7, and Us-8—were isolated from the stem and hook of Thai *U. attenuata*, which are exclusive to this species [20,21] (Figure S2b). These alkaloids with an opened D-ring were formed by an oxidative cleavage at the enamine double bond (C20–C21) of strictosidine aglycone intermediate during the biosynthesis process [21]. Yet, the potential neuroprotective activities of *U. attenuata* and its alkaloids are unknown. All these have sparked our interest in isolating and evaluating the cholinesterase-inhibitory activity of the alkaloid constituents from the leaves of Malaysian *U. attenuata*.

## 2. Materials and Methods

### 2.1. The General Experimental Procedures

Column chromatography was carried out on silica gel (230–400 mesh; Merck, Darmstadt, Germany). TLC (silica gel 60 F_254_, Merck, Darmstadt, Germany) was used to monitor fractions from column chromatography. Preparative TLC was performed on silica gel 60 F_254_ (20 cm × 20 cm, 0.5 mm; Merck, Darmstadt, Germany). Visualization of the TLC plates was achieved with a UV lamp (λ = 254 and 365 nm). Optical rotations were obtained utilizing a JASCO P-1010 polarimeter. Circular dichroism (CD) absorption spectra were recorded using a Jasco J-815 spectropolarimeter. ^1^H and ^13^C NMR spectra were obtained on Bruker model AMX 500 NMR spectrometers with standard pulse sequences, operating at 500 MHz in ^1^H and 125 MHz in ^13^C. The chemical shift values were reported in parts per million units (ppm) from trimethylsilane (TMS) using known solvent chemical shifts. Coupling constants were recorded in hertz (Hz). Standard pulse sequences were used for COSY, HMQC, HMBC, NOESY, and DEPT. High-resolution mass spectrum (HR-MS) was measured on a Waters Xevo G2-XS QTof quadrupole time-of-flight mass spectrometer (Waltham, MA, USA). Gas chromatography–mass spectra (GC-MS) were measured on an Agilent 6890 N Network GC system coupled to an Agilent 5973i mass selective detector (Agilent Technologies, Waldbronn, Germany). IR spectra were recorded by KBr using Perkin Elmer (Waltham, MA, USA) 2000 FT-IR spectrophotometer. Melting points were determined using a Stuart Scientific Melting Point SMP 1 (Staffordshire, UK) and were uncorrected. UV spectra were recorded on a Shimadzu UV-1800 spectrophotometer (Kyoto, Japan). The absorbance for AChE and BChE inhibitory assay was recorded on a Thermo Scientific Multiskan Go microplate reader (Waltham, MA, USA).

### 2.2. Chemicals and Reagents

Hexane, chloroform (CHCl_3_), ethyl acetate (EtOAc), and methanol (MeOH) used for the extraction and isolation of alkaloids were of analytical grade (Merck, Darmstadt, Germany). Reagents used for acid–base extraction were glacial acetic acid, anhydrous sodium sulfate, and ammonium hydroxide from Merck (Darmstadt, Germany). For the cholinesterase inhibitory assay, acetylthiocholine iodide and acetylcholinesterase from electric eel, bovine serum albumin, 5,5′-dithiobis (2-nitrobenzoic acid), and butyrylcholinesterase from equine serum, and S-butyrylthiocholine chloride and physostigmine were purchased from Sigma-Aldrich (St. Louis, MO, USA).

### 2.3. Plant Material

The leaves of *Uncaria attenuata* Korth. (Rubiaceae) were collected from Bukit Kledang, Ipoh, Perak, Malaysia (4°34′18.7763″ N, 101°1′39.4139″ E). The taxonomical identity of the plant was authenticated by Dr. Ooi Im Hin, a botanist from Penang Botanic Gardens, Malaysia. A voucher specimen (no. TAF 1) was then deposited at the herbarium of Penang Botanic Gardens.

### 2.4. Extraction of Plant Material

The air-dried and powdered leaf material (300 g) was extracted with MeOH (1:15 *w*/*v*) using a 5 L Soxhlet extractor for 48 h. The obtained supernatant was filtered and evaporated to dryness in vacuo to yield a crude MeOH extract (70 g). The MeOH extract was then subjected to acid–base extraction to enrich the alkaloid constituents. Briefly, the MeOH extract was partitioned between hexane (3 × 1 L) and 10% CH_3_COOH. The acidic aqueous layer was then adjusted to pH 9.0 with 25% NH_4_OH and extracted with CHCl_3_ (5 × 1 L). The CHCl_3_-soluble portion was washed with distilled water, dried over anhydrous sodium sulfate, and evaporated in vacuo to yield a crystalline alkaloid extract (2.5 g). 

### 2.5. Alkaloid Isolation and Identification

The alkaloid extract (2.5 g) was subjected to vacuum liquid chromatography on a silica gel (5 cm × 18 cm) using a step gradient of hexane-EtOAc-MeOH (50:50:0 to 0:0:100, *v*/*v*/*v*) to yield six fractions (Fr. 1–Fr. 6). F3 (200 mg) was further separated by using silica gel column chromatography (hexane-EtOAc-MeOH 20:80:0 to 0:90:10, *v*/*v*/*v*) to afford four subfractions (SFr. 3a, 3b, 3c, and 3d). SFr. 3b (20 mg) was then purified using preparative TLC (EtOAc-MeOH 9:1, *v*/*v*) to yield villocarine A (**2**) (8 mg; TLC R_f_ 0.50) and geissoschizine methyl ether (**3**) (3 mg; TLC R_f_ 0.63). Next, Fr. 4 (200 mg) was repeatedly purified by silica gel CC to afford isovillocarine D (**1**) (9 mg). Spectroscopic and spectrometric data for the known compounds (**2**) and (**3**) agreed with that reported in the literature [22,23]. 

Isovillocarine D (**1**): white amorphous powder; [α]$\begin{array}{c} 18\\ D \end{array}$ +17.1 (*c* 0.00058, MeOH); mp 157.5–150.0 °C; +HRESI-MS: *m*/*z* 383.1830 [M + H]^+^, calculated for C_22_H_27_N_2_O_4_, 383.1893; UV λ_max_ (nm): 204.5, 243.0, 288.0. IR (film, KBr) ν_max_ 3254, 2946, 1708, 1639, 1621, 1471, 1246, 1146 cm^−1^; ^1^H and ^13^C NMR data, see Table 1. 

### 2.6. Cholinesterase Inhibitory Assay

The cholinesterase-inhibitory potential of the extracts and isolated compounds was determined using the spectrophotometric method described by Ellman et al. (1961) [24]. The assay procedure was the same as reported in our previous publications [25,26].

### 2.7. Molecular Docking

Molecular docking was performed only for the most active compound—villocarine A (**2**)—using Autodock 3.0.5 (La Jolla, CA, USA) along with AutoDockTools (ADT) to get insight into the molecular interactions and bonding affinities of the molecule in the active sites of the AChE and BChE enzymes following the method described in our previous publication [25]. Compound **2** was built using Hyperchem 8, and energy minimization was performed with a convergence criterion of 0.05 kcal/(mol A). The proper protein crystal structures of AChE from *Torpedo californica* in complex with galanthamine (PDB ID: 1W6R) and BChE from *Homo sapiens* (PDB ID: 2WIJ) were obtained from Protein Data Bank. For each docking experiment, one hundred independent dockings were carried out, and the lowest docked energy of each conformation in the most populated cluster was selected.

## 3. Results and Discussion

### 3.1. Structure Elucidation of Uncaria Attenuata Alkaloids **1**–**3**

**Figure 1:**
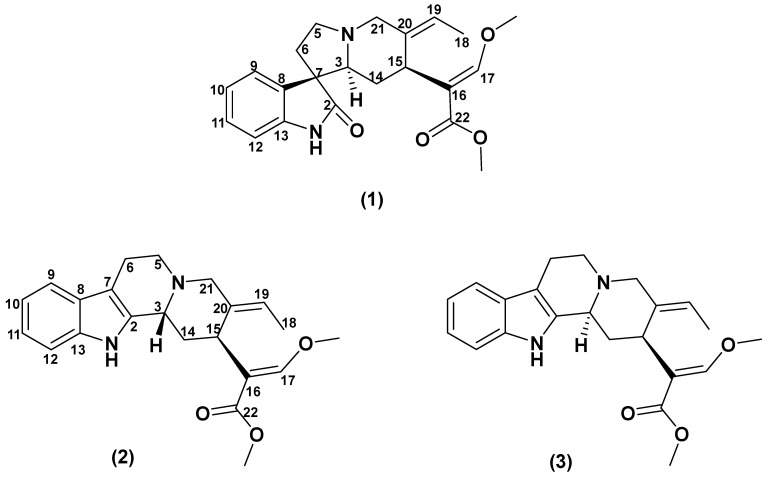
Chemical structure of compounds (**1**–**3**) isolated from the leaves of Malaysian *Uncaria attenuata*.

Screening data showed that the *U. attenuata* leaf extract exhibited potential inhibitory action against AChE and BChE enzymes with IC_50_ values below 50 µg/mL (Table 2). The alkaloid constituents were then enriched using acid–base extraction method and subjected to multiple-column chromatography to afford an undescribed oxindole alkaloid—isovillocarine D (**1**)—and two known indole alkaloids—villocarine A (**2**) and geissoschizine methyl ether (**3**). The chemical structure of these alkaloids was established based on multiple spectroscopic methods, such as UV, IR, ECD, EI-MS, ESI-MS, and NMR (Figure 1). The spectroscopic data of compounds (**1**–**3**) are provided in Table 1 and Supplementary Data (Figures S3–S21 and Tables S1 and S2). The stereochemical configuration of (**2**) at C3 and C15 were confirmed as *R* and *S*, respectively, based on NOE correlations and biogenesis considerations, as well as ECD analysis. The ECD spectrum of (**2**) showed a negative Cotton effect at 250–300 nm corresponding to the *β* orientation at H-3 (i.e., speciociliatine) (Figure S18) [27]. Compound (**3**) was identified as geissoschizine methyl ether, which is the 3*S* epimer of (**2**) based on the deshielded C3 signal at δ_c_ 58.9 (corresponded to δ_H_ 3.56) compared to (**2**) (δ_c_ 56.0, corresponded to δ_H_ 3.72) (Tables S1 and S2) [27,28]. This was further supported by the EI-MS fragmentation pattern of (**3**) (Figure S19), in which the M-CH_3_ fragment (*m*/*z* 351) was less intense than the M^+^ ion (*m*/*z* 366), in contrast to the EI-MS fragments observed for (**2**) (3*R*) (Figure S14) [28,29]. Notably, compounds (**1**–**3**) were reported for the first time in the leaves of *U. attenuata* native to Malaysia. The isomeric compounds (**2**) and (**3**) can also be found in the leaves of *Uncaria villosa* and the hooks of *Uncaria rhynchophylla* [22,30].

**Figure 2:**
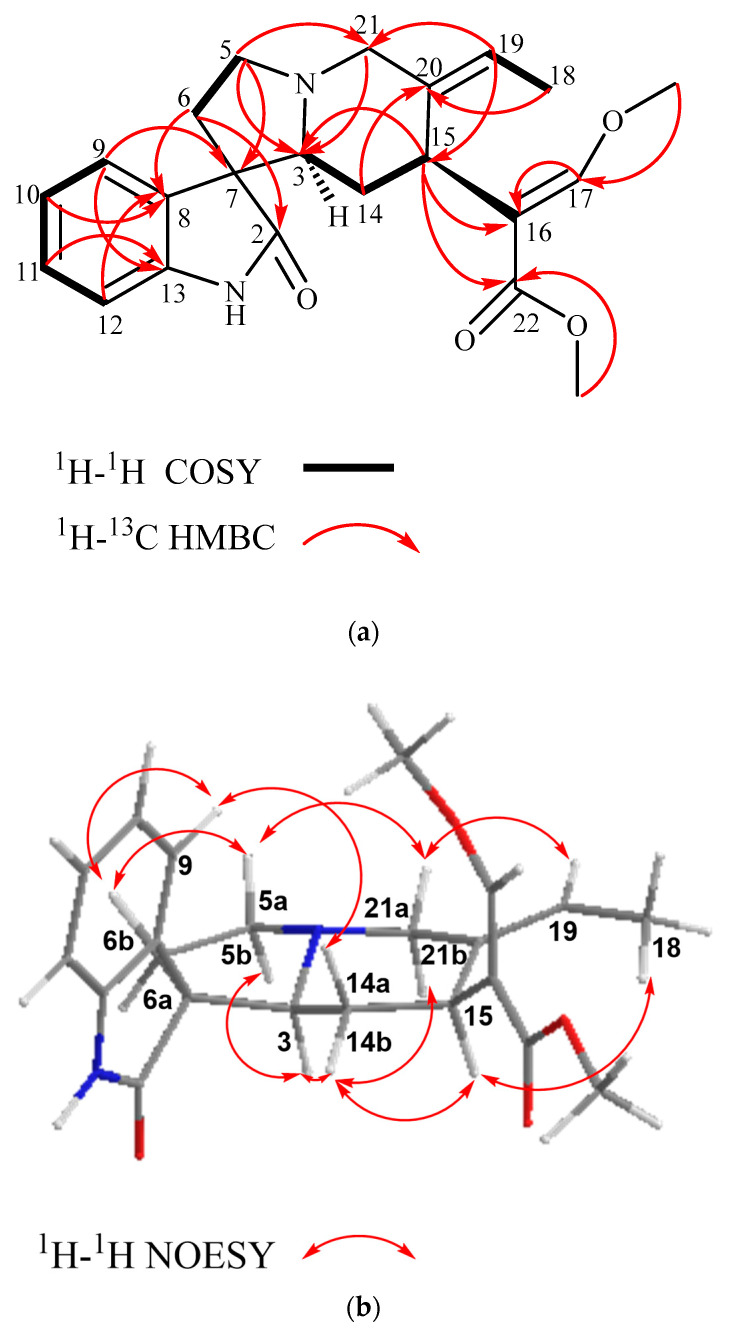
(**a**) Selected COSY and HMBC and (**b**) selected NOE correlations of compound (**1**) (most stable conformation, Chemdraw3D).

Isovillocarine D (**1**) was isolated as a white amorphous powder. Compound (**1**) had the molecular formula C_22_H_26_N_2_O_4_ deduced from the ^13^C NMR data and a pseudo molecular ion at *m*/*z* 383.1830 [M + H]^+^ (calculated for C_22_H_27_N_2_O_4_, 383.1893) in the HR-ESIMS, which was in consistency with 11 degrees of unsaturation. The ^1^H NMR data (Table 1) indicated compound (**1**) possessed an oxindole moiety with an indolic proton (NH) at δ_H_ 7.48 (1H, s); an ortho-disubstituted aromatic system at δ_H_ 7.41 (1H, br d, *J* = 7.8 Hz, H-9), 6.83 (1H, br. d, *J* = 7.8 Hz, H-12), 7.19 (1H, td, *J* = 7.8, 1.0 Hz, H-11), and 7.02 (1H, td, *J* = 7.8, 1.0 Hz, H-10) in conjunction with the ^13^C NMR data (δ_C_ 181.5 (C-2), 139.9 (C-13), 133.1 (C-8), 127.5 (C-11), 125.5 (C-9), 122.4 (C-10), and 56.7 (C-7)); and a *β*-methoxy acrylate moiety at δ_H_ 7.25 (1H, s, H-17), 3.66 (3H, *s*, OCH_3_-22), and 3.76 (3H, s, OCH_3_-17) in conjunction with the ^13^C NMR signals at δ_C_ 168.7 (C-22), 158.7 (C-17), 111.9 (C-16), 61.4 (OCH_3_-17), and 51.3 (OCH_3_-22). The above signals, including the typical carbonyl carbon at δ_C_ 181.5 (C-2) (Figure 2a), suggested that compound (**1**) is a corynanthe-type oxindole alkaloid like that of corynoxine isolated from *Mitragyna speciosa* [27,31]. The ^13^C NMR and HSQC data (Table 1 and Figures S7 and S12) indicated that compound (**1**) had 22 carbon signals, including the above-mentioned carbon signals, and an additional 9 carbon signals: 4 methylenes (δc 60.4 (C-21), 54.6 (C-5), 35.4 (C-6), and 31.8 (C-14)), 2 methines (δ_C_ 68.4 (C-3) and 30.5 (C-15)), 1 methyl (δ_C_ 13.1 (C-18)), and 1 extra double bond (δ_C_ 135.1 (C-20) and 121.8 (C-19)). A comparison of ^1^H and ^13^C NMR spectra of both (**1**) and corynoxine (Figure S2a) indicated that the typical signals of an ethyl side chain at position C-20 were missing in (**1**) [28,29]. However, a Δ^19(20)^ olefinic moiety and a deshielded methyl group can be observed at δ_H_ 5.41 (q, *J* = 6.7 Hz, H-19); δ_C_ 121.8 (C-19) and 135.1 (C-20) and δ_H_ 1.52 (dd, *J* = 6.8, 1.8 Hz, H-18); δ_C_ 13.1 (C-18), suggesting the presence of an ethylidene side chain in (**1**) [22,23]. The assigned ^1^H and ^13^C NMR data of (**1**) were further supported by the 2D NMR experiments: ^1^H-^1^H COSY and HMBC (Figure 2a). The location of the ethylidene side chain was established by the HMBC correlations observed for H-19 with C-15, C-18, and C-21 and H-18 with C-20 and C-19. The HMBC correlations of H-15 with C-16, C-17, and C-22 indicated that the *β*-acrylate chain was substituted at position 15, which is one of the characteristic features of corynanthe-type alkaloids [31].

The configuration of (**1**) at C-3, C-7, and C-15 can be readily deduced from the 2D NOESY experiment (Figure 2b). In the experiment, H-15 (δ_H_ 3.84) showed spatial correlations with CH_3_-18 (δ_H_ 1.52) and H-14b (δ_H_ 1.28) and, subsequently, H-14b with H-3 (δ_H_ 2.75), and H-3 with H-21b (δ_H_ 3.32) and H-5b (δ_H_ 2.52). Following the biogenesis theory of corynanthe-type alkaloids, H-15 naturally occurs in the *α*-orientation, thus, indicating that all the above-correlated protons were on the same plane (*α*-orientation) [32,33,34]. The NOE correlation of H-15 with H_3_-18 also suggested that the geometry of the ethylidene side chain was in the (*E*)-configuration [23]. On the other hand, H-5a (δ_H_ 3.36) exhibited NOE correlations with H-6b (δ_H_ 2.08) and H-21a (δ_H_ 3.54) and, subsequently, H-21a with H-19 (δ_H_ 5.41), indicating these correlated protons were in the *β*-orientation. The relative configuration of the spiro-carbon, C-7, was assigned to be *S* based on the NOE correlations of H-9 with H-14*β* (δ_H_ 1.32) and H-6*β* (δ_H_ 2.08), which indicated that both H-14*β* and H-6*β* of (**1**) were located within the shielding zone of the aromatic ring [35] (Figure 2b). Therefore, the structure of isovillocarine D (3*S*,7*S*,15*S*) was established as (**1**) (Figure 1), which is an undescribed 7*S*-epimer of villocarine D isolated from *Uncaria villosa* [23]. This proposed structure of (**1**) was further supported by the downshift of C-3 (δc 68.4) as well as the upshift of H-14*β* by 1.2 ppm (δ_H_ 1.32) compared to villocarine D (7*R*) (H-14*β*, δ_H_ 2.52; C-3, δc 65.8) [23,35].

### 3.2. Cholinesterase-Inhibitory Activity

The total alkaloid extract of *U. attenuata* and the two major alkaloids, isovillocarine D (**1**) and villocarine A (**2**), and the minor alkaloid, geissoschizine methyl ether (**3**), were evaluated for their inhibition against AChE and BChE enzymes. The total alkaloid extract inhibited both enzymes with IC_50_ values of 8.90 and 21.74 µg/mL, respectively, which were about two times more potent than the origin methanolic extract (Table 2). Among the tested alkaloids, compound (**2**) displayed the most potent inhibitory effect against both AChE and BChE with IC_50_ values of 14.45 and 13.95 µM, respectively, regarded as a dual cholinesterase inhibitor (selectivity index ≈ 1). On the other side, compounds (**3**) and (**1**) were found to be moderate but selective BChE inhibitors (IC_50_ 17.65 and 35.28 µM, respectively). The dose-dependent curve of extracts and individual alkaloids against AChE and BChE are provided in the Supplementary Materials (Supplementary Figures S22 and S23). Surprisingly, all the isolated compounds showed better selectivity toward BChE than AChE, which differed from the methanolic and alkaloid-enriched extracts. This suggested that the anti-AChE activity of *U. attenuata* extracts was possibly due to the synergistic effects among the alkaloid constituents. 

A closer study of their structure–inhibition correlations revealed that the H-3*β* position at the indole C/D ring of (**2**) is critical for AChE inhibition, as evidenced by the higher IC_50_ value observed for its 3*S*-epimer (**3**), which was about three times higher than (**2**). Remarkably, a loss of anti-AChE activity was observed when the indole ring (**3**) was oxidized to form the spirocyclic oxindole (**1**), suggesting that the indole moiety is essential for inhibiting the enzyme. For BChE, both compounds (**2**) and (**3**) inhibited the enzyme with similar IC_50_ values, demonstrating that the chirality at C-3 has little to no effect on BChE inhibition. Like the AChE enzyme, the anti-BChE activity of (**1**) was cut in half compared to the IC_50_ value of (**3**). This showed that indole moiety is also preferable for BChE inhibition. The lack of anticholinesterase activity in corynanthe-type oxindole alkaloids was supported by other published data, in which rhynchophylline, isorhynchophylline, corynoxeine, and isocorynoxeine had no inhibition on AChE and BChE enzymes, even when tested at 100 µg/mL [36,37,38].

### 3.3. Molecular Docking Study

**Figure 3:**
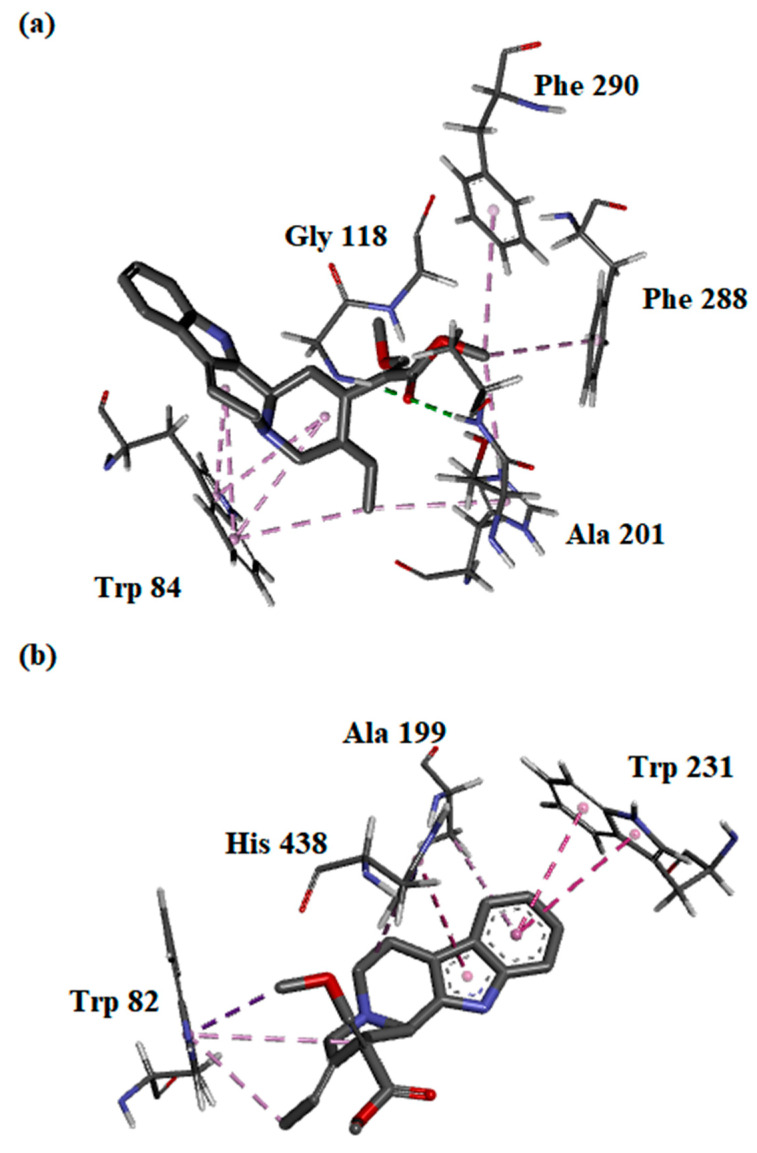
Binding orientations and interactions of compound (**2**) with protein residues at the active site of (**a**) acetylcholinesterase and (**b**) butyrylcholinesterase.

The binding interactions between the most potent compound—compound (**2**)—and both AChE and BChE enzymes were evaluated in silico. The free energy of binding (FEB) of (**2**) with AChE was slightly higher than BChE, which correlated with the IC_50_ values obtained (Table 3). In silico analysis showed that the *β*-methoxy acrylate moiety of (**2**) interacted with key amino acids of AChE at the oxyanion hole (Gly 118 and Ala 201) via hydrogen bonding. Further, (**2**) was predicted to interact with the key residues of the choline-binding site and acyl-binding pocket of AChE through hydrophobic interactions. Multiple π–alkyl interactions were observed between Trp 84 with indole C-ring, D-ring, and the C_20_-ethylidene chain of (**2**), which further substantiates the importance of the indole moiety and C_20_-ethylidene group in inhibiting AChE (Figure 3a). On the other hand, only the hydrophobic interaction of (**2**) with the choline-binding site, catalytic side, and oxyanion hole may explain the higher FEB with BChE. Similar to AChE, the interactions of (**2**) with the key amino acids within the BChE pocket were mainly established through its indole moiety and C_20_-ethylidene group (Figure 3b). 

## 4. Conclusions

The present study reported the cholinesterase-inhibitory activity of *U. attenuata* and its alkaloid constituents for the first time. A new corynanthe-type oxindole alkaloid and two known indole alkaloids were isolated and characterized from the leaves of Malaysian *U. attenuata*. The new oxindole alkaloid was identified as isovillocarine D (3*S*,7*S*,15*S*) (**1**) using various modern spectroscopic analyses. All the isolated compounds (**1**–**3**) showed moderate to weak cholinesterase inhibition with IC_50_ values lower than 50 µM. Compound (**2**) had the most potent inhibitory activity against both AChE and BChE, with IC_50_ values of 14.45 and 13.94 µM, respectively, followed by compounds (**3**) and (**1**). In terms of selectivity, compound (**2**) is a dual inhibitor (selectivity ≈ 1), whereas (**1**) and (**3**) are selective BChE inhibitors. Molecular docking studies showed that (**2**) interacted with the active site of AChE and BChE mainly by forming hydrogen and hydrophobic bonds between the key amino acids and the indole moiety and ethylidene side chain. Overall, corynanthe-type indole alkaloids from *U. attenuata* showed potential inhibitory activity on AChE and BChE. The plant could be a good source for searching for new cholinesterase inhibitors.

## Figures and Tables

**Table 1:** 1D and 2D NMR data of compound (**1**).

Compound (1)				
Position	^1^H [δ_H_ (*J*, Hz)]	^13^C (δc)	HMBC ^1^H to ^13^C	NOESY ^1^H to ^1^H
NH	7.48 [br s (1H)]	-	7, 8	-
2	-	181.5	-	-
3	2.75 [dd (1H, 11.9, 2.6)]	68.4	8	5b, 14b, 21b
5a	3.36 [overlapped (1H)]	54.6	3, 7	6b, 21a
5b	2.52 [q (1H, 8.9)]		6, 21	3
6a	2.42 [ddd (1H, 9.2, 2.2)]	35.4	2, 5	-
6b	2.08 [m (1H)]		8	5a, 9
7	-	56.7	-	-
8	-	133.1	-	-
9	7.41 [br d (1H, 7.8)]	125.5	7, 11, 13	6b, 14a
10	7.02 [td (7.8, 1.0)]	122.4	8, 12	-
11	7.19 [td (1H, 7.8, 1.0)]	127.5	9, 13	-
12	6.83 [br d (1H, 7.8)]	109.1	8, 10	-
13	-	139.9	-	-
14a	1.32 [s (1H)]	31.8	6, 15, 16	-
14b	1.28 [s (1H)]		3, 5, 15, 20	3, 15, 21b
15	3.84 [d (1H, 6.9)]	30.5	3, 14, 16, 17, 19, 20, 21, 22	14b, 18, 21b
16	-	111.9	-	-
17	7.25 [s (1H)]	158.7	15, 16, OCH_3_-17, 22	OCH_3_-17
18	1.52 dd [(3H, 6.8, 1.8)]	13.1	19, 20	15
19	5.41 [q (1H, 6.7)]	121.8	15, 18, 21	21a
20	-	135.1	-	-
21a	3.54 [br d (1H, 12.4)]	60.4	3, 15, 19, 20	5a, 19
21b	3.32 [overlapped (1H)]		3, 19, 20	3, 14b, 5b
22	-	168.7	-	-
OCH_3_-22	3.66 [s (3H)]	51.3	22	-
OCH_3_-17	3.76 [s (3H)]	61.4	17	17

**Table 2:** Cholinesterase-inhibitory activity of *Uncaria attenuata* and its isolated compounds (**1**–**3**).

Samples	AChE			BChE			Selectivity Index	
	% Inhibition at 100 µM	IC_50_, µM	IC_50_, µg/mL	% Inhibition at 100 µM	IC_50_, µM	IC_50_, µg/mL	AChE ^a^	BChE ^b^
Methanol extract *	-	-	16.46 ± 2.30	-	-	46.32 ± 1.17	2.81	0.36
Alkaloid extract *	-	-	8.90 ± 2.43	-	-	21.74 ± 4.34	2.44	0.41
Compound (**1**)	22.11 ± 3.23	-	-	87.44 ± 1.34	35.38 ± 5.60	13.51	-	-
Compound (**2**)	75.55 ± 2.61	14.45 ± 2.94	5.29	95.80 ± 2.33	13.94 ± 2.69	5.10	0.97	1.04
Compound (**3**)	81.53 ± 1.45	40.55 ± 0.91	14.84	90.75 ± 3.56	17.64 ± 0.58	6.45	0.43	2.30
Galantamine HBr	-	0.94 ± 0.12	0.35	-	30.41 ± 1.11	11.20	32.35	0.03

All data are expressed as mean ± standard deviation (*n* = 3). * IC_50_ values of extracts are expressed as µg/Ml. ^a^ Selectivity for AChE is defined as IC_50_(BChE)/IC_50_(AChE). ^b^ Selectivity for BChE is defined as IC_50_(AChE)/IC_50_(BChE).

**Table 3:** Binding interaction data for compound (**2**) docked into active site gorge of *T*cAChE and *h*BChE.

	Binding Energy (kcal)	Residue	Type of Interaction	Distance (Å)	Interaction Sites
*Tc*AChE	−13.40	Trp 84	Pi–alkyl	4.40	Choline-binding site
			Pi–alkyl	5.00	
			Pi–alkyl	5.30	
		Gly 118	H-bond	1.98	Oxyanion hole
		Ala 201	H-bond	2.69	Oxyanion hole
		Phe 290	Pi–alkyl	4.74	Acyl-binding pocket
		Phe 288	Pi–alkyl	4.96	Acyl-binding pocket
*h*BChE	−11.21	Trp 82	Pi–sigma	3.68	Choline-binding site
			Pi–alkyl	5.22	
			Pi–alkyl	4.17	
		His 438	Pi-alkyl	5.46	Catalytic site
			Pi-cation	5.76	
		Ala 199	Pi-alkyl	4.65	Oxyanion hole

## Data Availability

The data presented in this study are available in the article and Supplementary Materials.

## Supplementary Materials

The following supporting information can be downloaded at: https://www.mdpi.com/article/10.3390/metabo13030390/s1, Figure S1: Examples of clinically approved cholinesterase inhibitors; Figure S2: (a) Common corynanthe-type neuroprotective alkaloids found in *Uncaria* species; (b) unusual D-secocorynanthe-type oxindole alkaloids from the stems and hooks of Thai *Uncaria attenuata*; Figure S3: UV spectrum of compound (**1**); Figure S4: Q-ToF-ESI-MS spectrum of compound (**1**); Figure S5: IR spectrum of compound (**1**); Figures S6–S13: 1D and 2D NMR spectra of compound (**1**); Figure S14: EI-MS spectrum of compound (**2**); Figures S15–S16: 1D NMR spectra of compound (**2**); Figure S17: EI-MS spectrum of compound (**3**); Figure S18: ECD spectrum of compound (**2**); Figure S19: EI-MS spectrum of (**3**); Figures S20–S21: 1D NMR spectra of compound (**3**); Figure S22: The dose-dependent curve of (a) methanol and alkaloid extracts, and (b) compounds (**2**) and (**3**) against acetylcholinesterase (AChE); Figure S23: The dose-dependent curve of (a) methanol and alkaloid extracts, and (b) compound (**1**–**3**) against butyrylcholinesterase (BChE). Table S1: 1D NMR data of compound (**2**); Table S2: 1D NMR data of compound (**3**).
