# Supplementary material for: Natural Corynanthe-Type Cholinesterase Inhibitors from Malaysian Uncaria attenuata Korth.: Isolation, Characterization, In Vitro and In Silico Studies
Source: Metabolites. 2023 Mar 7;13(3):390. doi: 10.3390/metabo13030390 (PMC10059728; doi:10.3390/metabo13030390)
Supplement: Supplementary file 1 [file metabolites-13-00390-s001.zip › metabolites-2165917-supplementary.pdf]

Supplementary Materials

Natural Corynanthe-Type Cholinesterase Inhibitors from Malaysian *Uncaria attenuata* Korth.: Isolation, Characterization, *In vitro* and *In silico* Studies

Nelson Jeng-Yeou Chear [1†], Tan Ai Fein Ching-Ga [1†], Kooi-Yeong Khaw[2], Francisco León[3], Wen-Nee Tan[4], Siti R. Yusof[1], Christopher R. McCurdy[5], Vikneswaran Murugaiyah[1,6]*, and Surash Ramanathan[1]*

[1]*Centre for Drug Research, Universiti Sains Malaysia, 11800 Minden, Penang, Malaysia*

[2]*School of Pharmacy, Monash University Malaysia, 47500 Bandar Sunway, Selangor, Malaysia*

[3]*Department of Drug Discovery and Biomedical Sciences, College of Pharmacy, University of South Carolina, Columbia, SC 29201, United States*

[4]*Chemistry Section, School of Distance Education, Universiti Sains Malaysia, 11800 Minden, Penang, Malaysia*

[5]*Department of Medicinal Chemistry, College of Pharmacy, University of Florida, Gainesville, FL 32610, United States*

[6]*Discipline of Pharmacology, School of Pharmaceutical Sciences, Universiti Sains Malaysia, 11800 Minden, Penang, Malaysia.*

*Corresponding authors:

Surash Ramanathan (PhD), Centre for Drug Research, Universiti Sains Malaysia, 11800 Minden, Penang, Malaysia. Email: srama@usm.my

Vikneswaran Murugaiyah (PhD), Centre for Drug Research, Universiti Sains Malaysia, 11800 Minden, Penang, Malaysia. Email: vicky@usm.my

† These authors equally contributed.

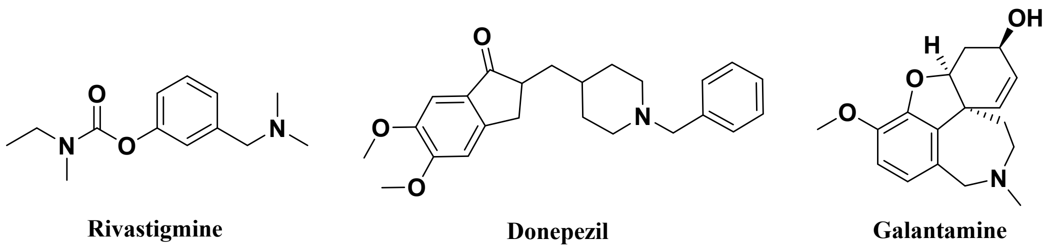

Figure S1. Examples of clinically approved cholinesterase inhibitors.

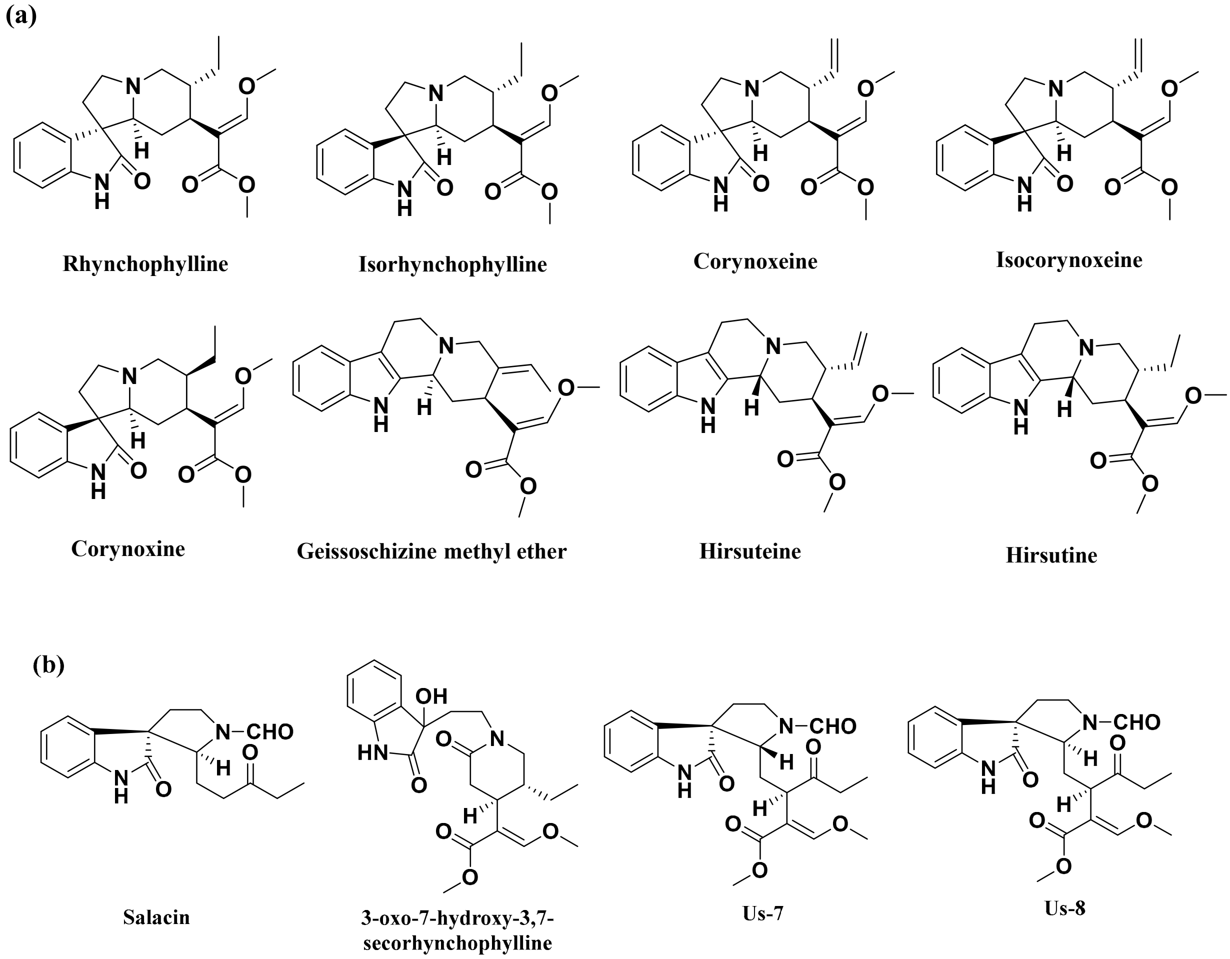

Figure S2. (**a**) Common corynanthe-type neuroprotective alkaloids found in *Uncaria* species; (**b**) Unusual D-secocorynanthe-type oxindole alkaloids from the stems and hooks of Thai *Uncaria attenuata*.

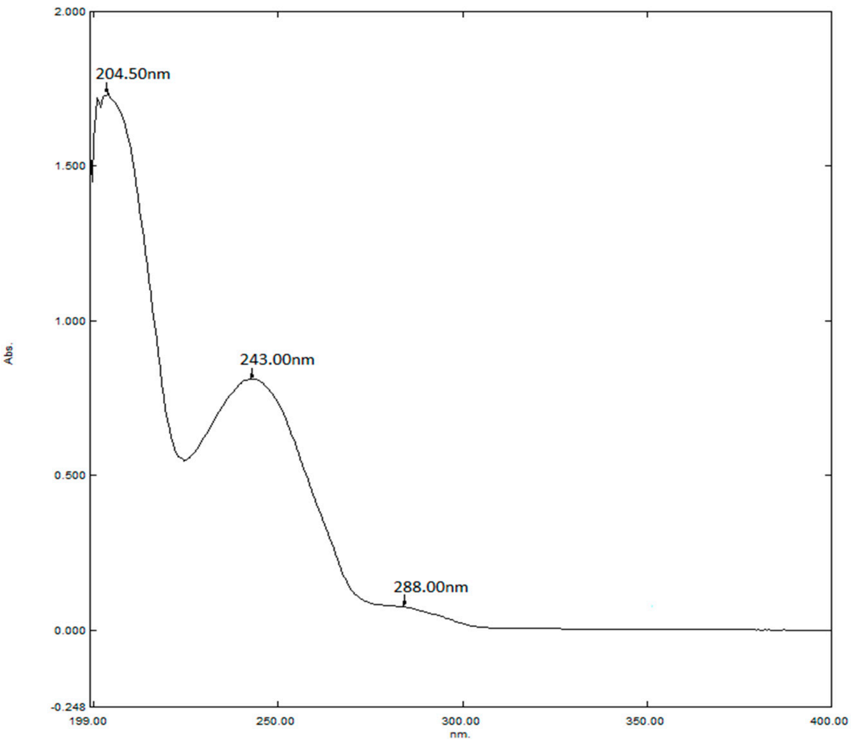

Figure S3: UV spectrum of compound (**1**) (MeOH).

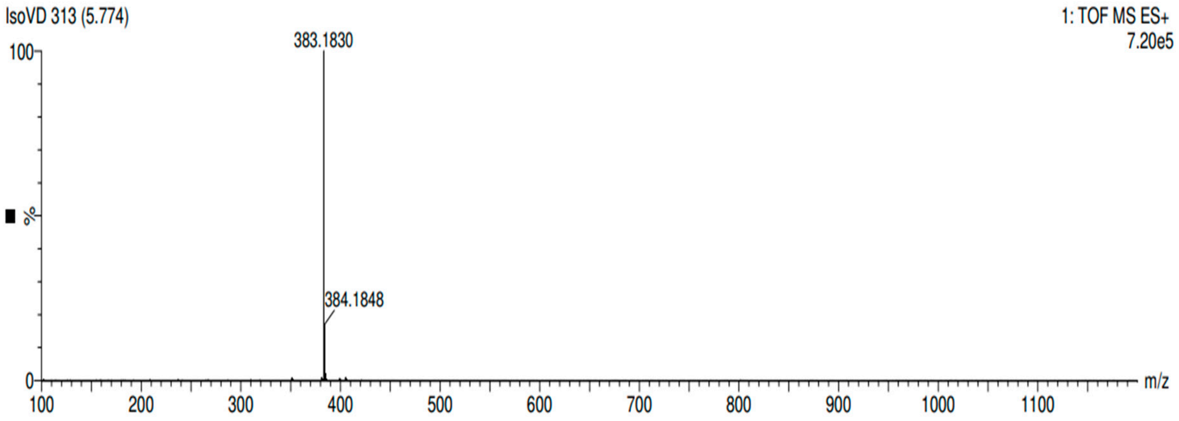

Figure S4: QToF-MS spectrum of compound (**1**) (positive mode).

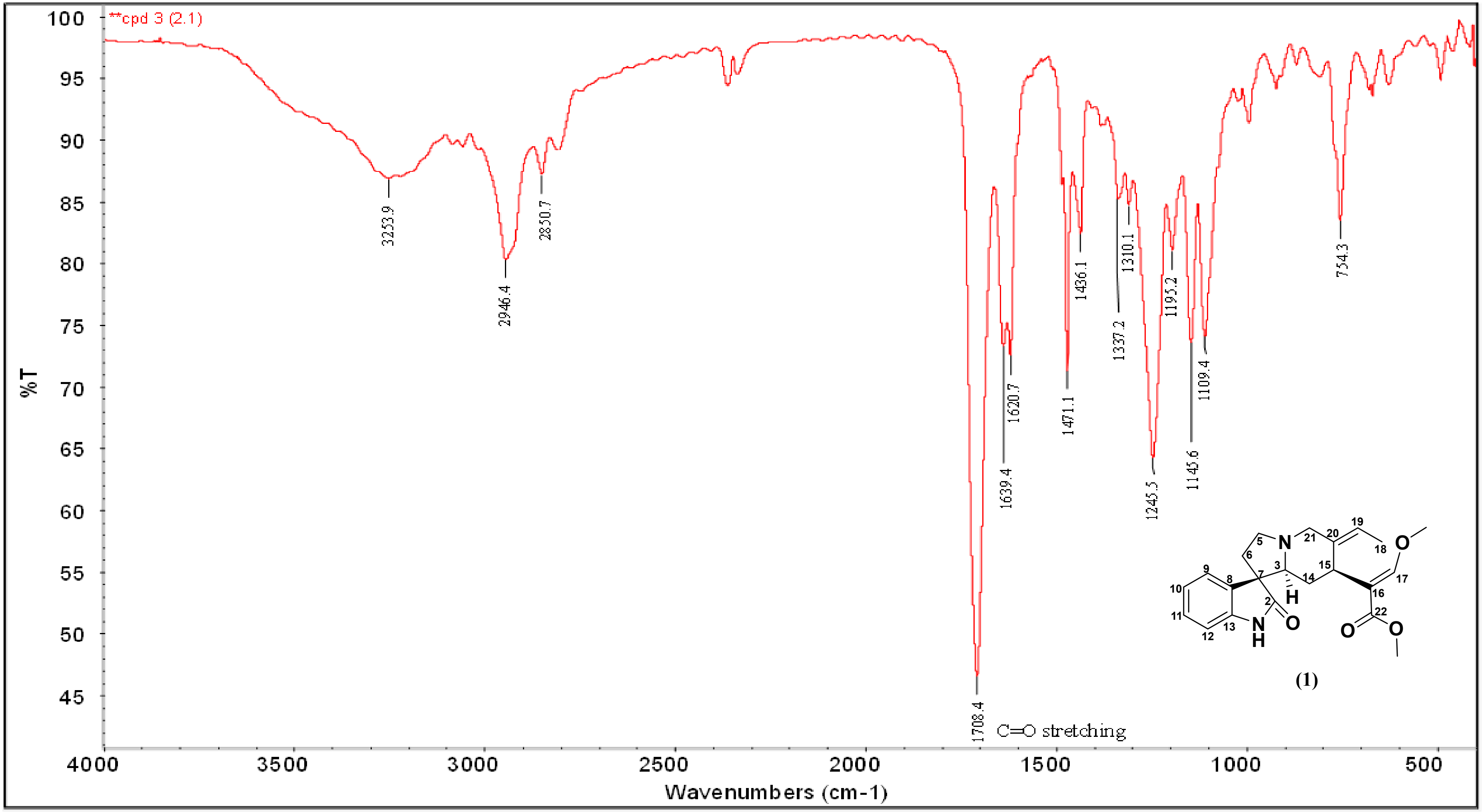

Figure S5: FTIR spectrum of compound (**1**) (KBr).

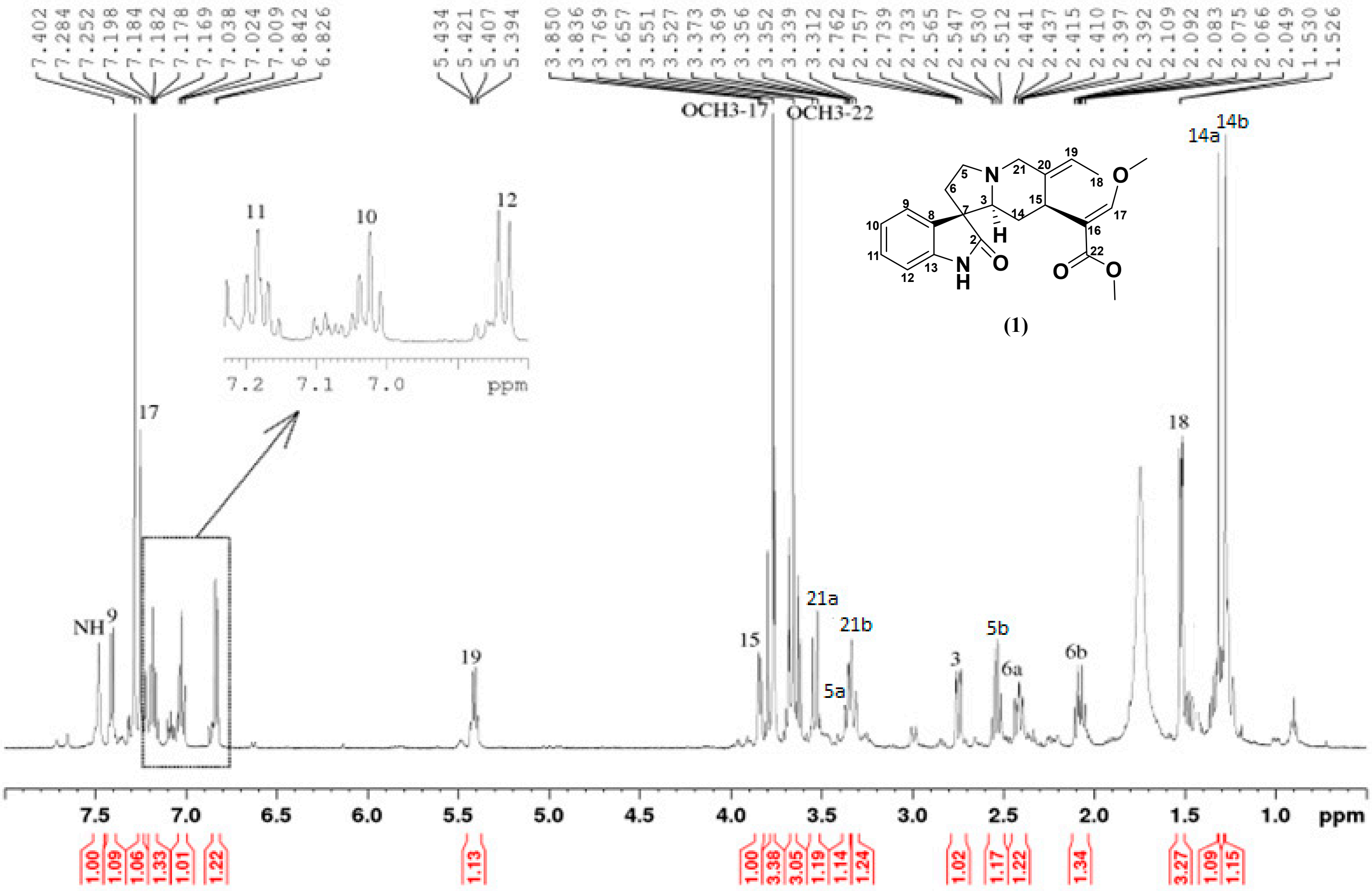

Figure S6: ^{1}H NMR spectrum of compound (**1**) ($CDCl_3$, 500MHz).

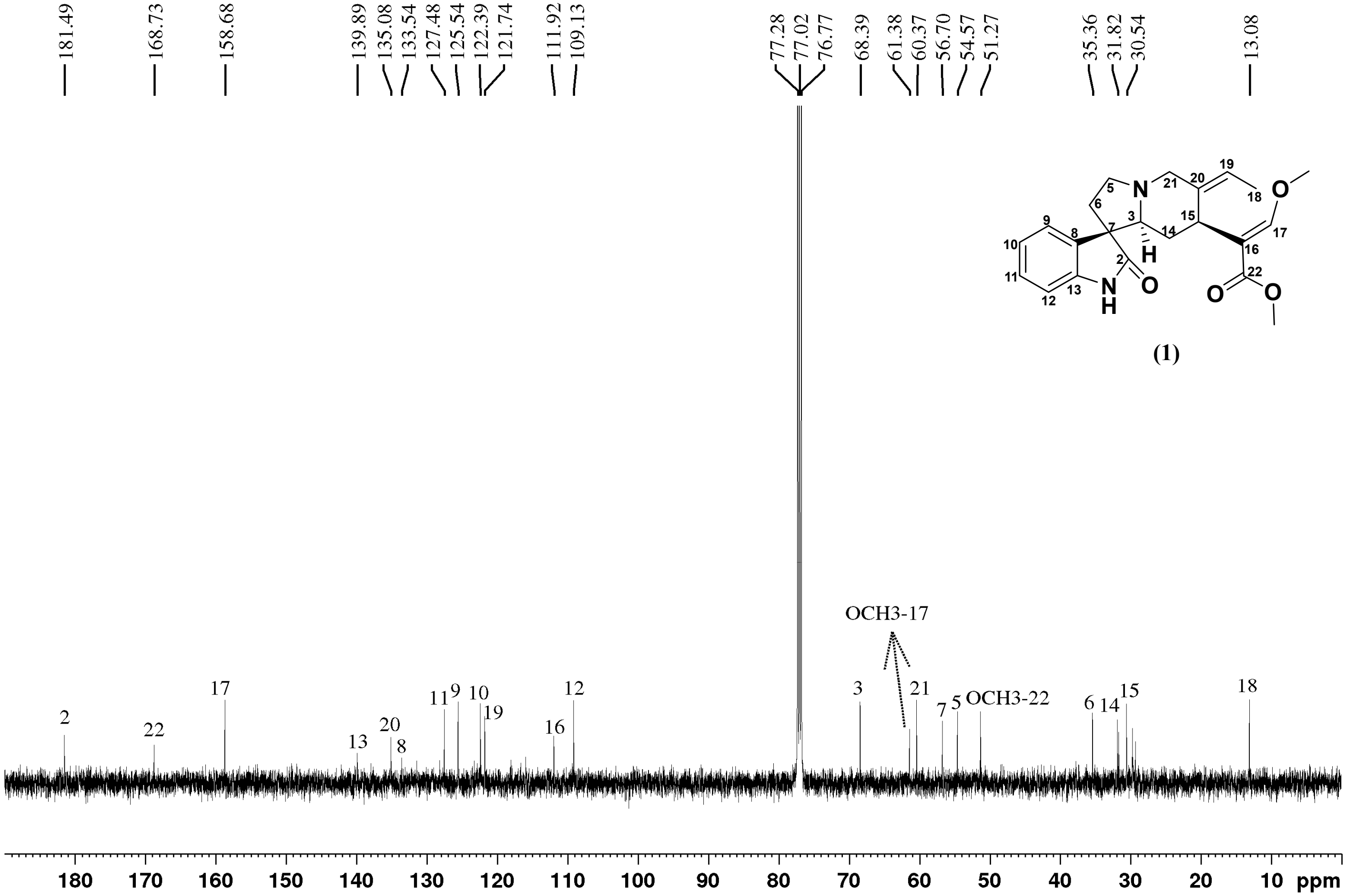

Figure S7: ^{13}C NMR spectrum of compound (**1**) ($CDCl_3$, 125MHz).

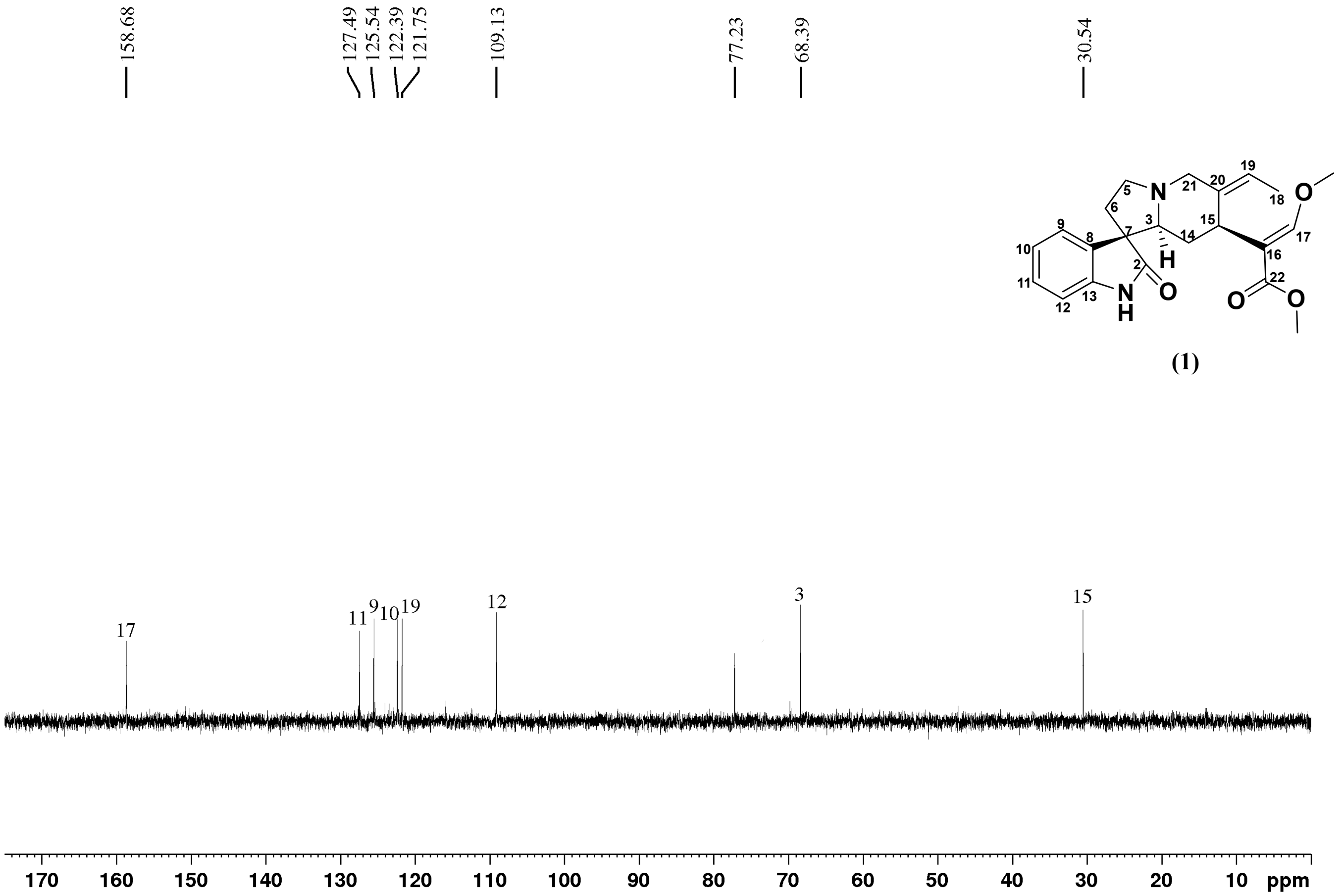

Figure S8: DEPT 90 spectrum of compound (**1**) ($CDCl_3$, 125MHz).

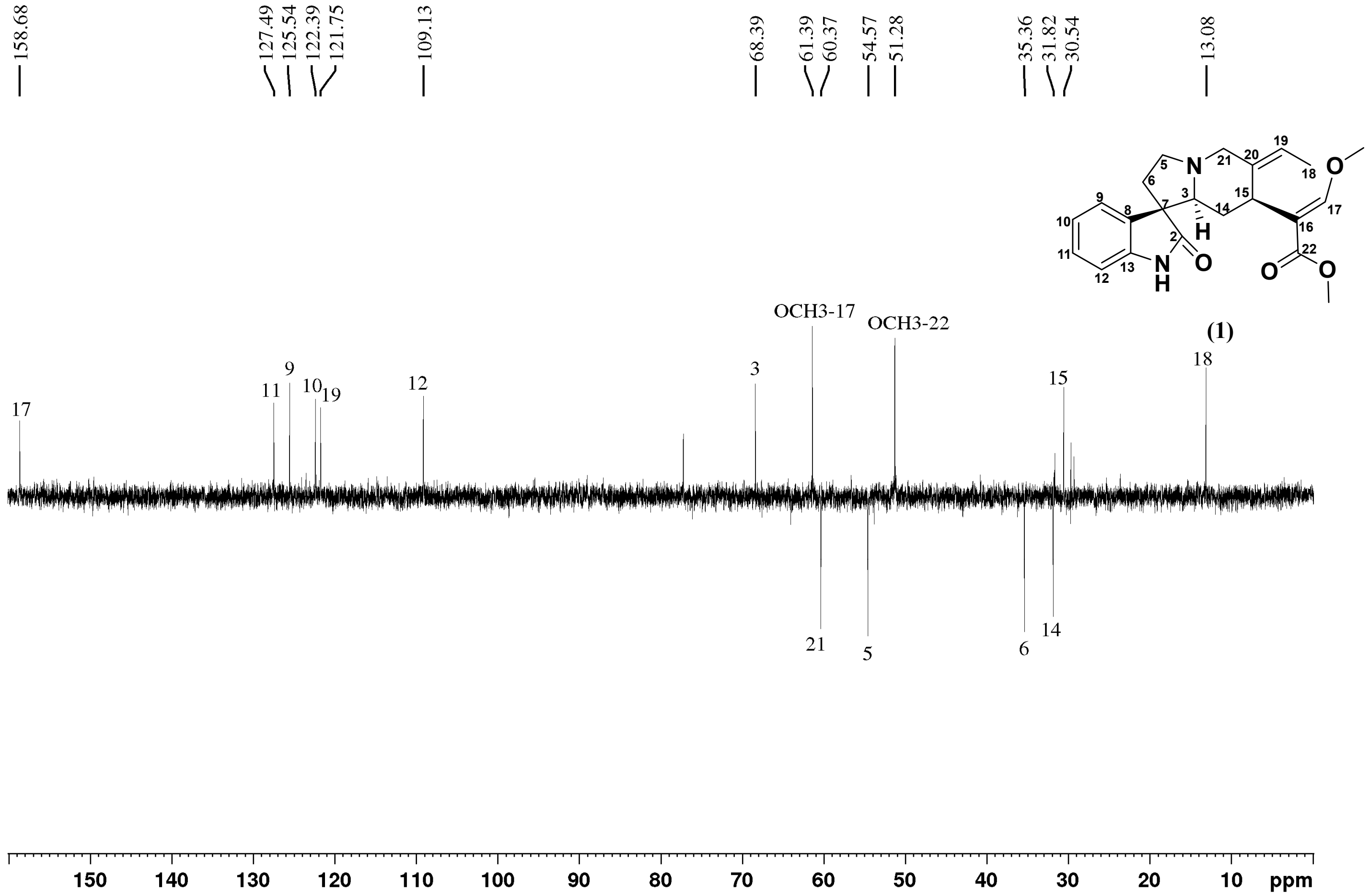

Figure S9: DEPT 135 spectrum of compound (**1**) ($CDCl_3$, 125MHz).

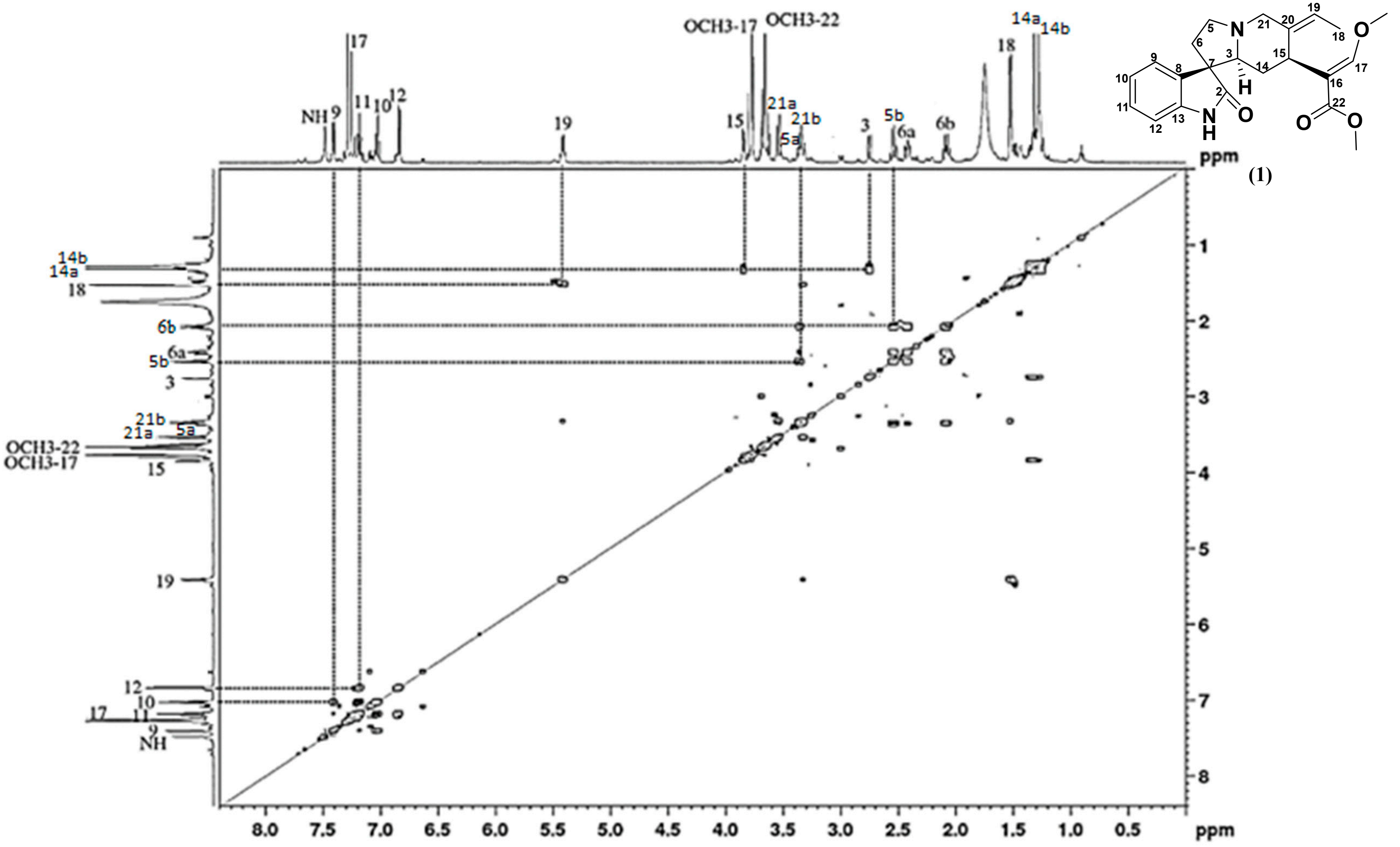

Figure S10: COSY spectrum of compound (**1**) ($CDCl_3$, 500MHz).

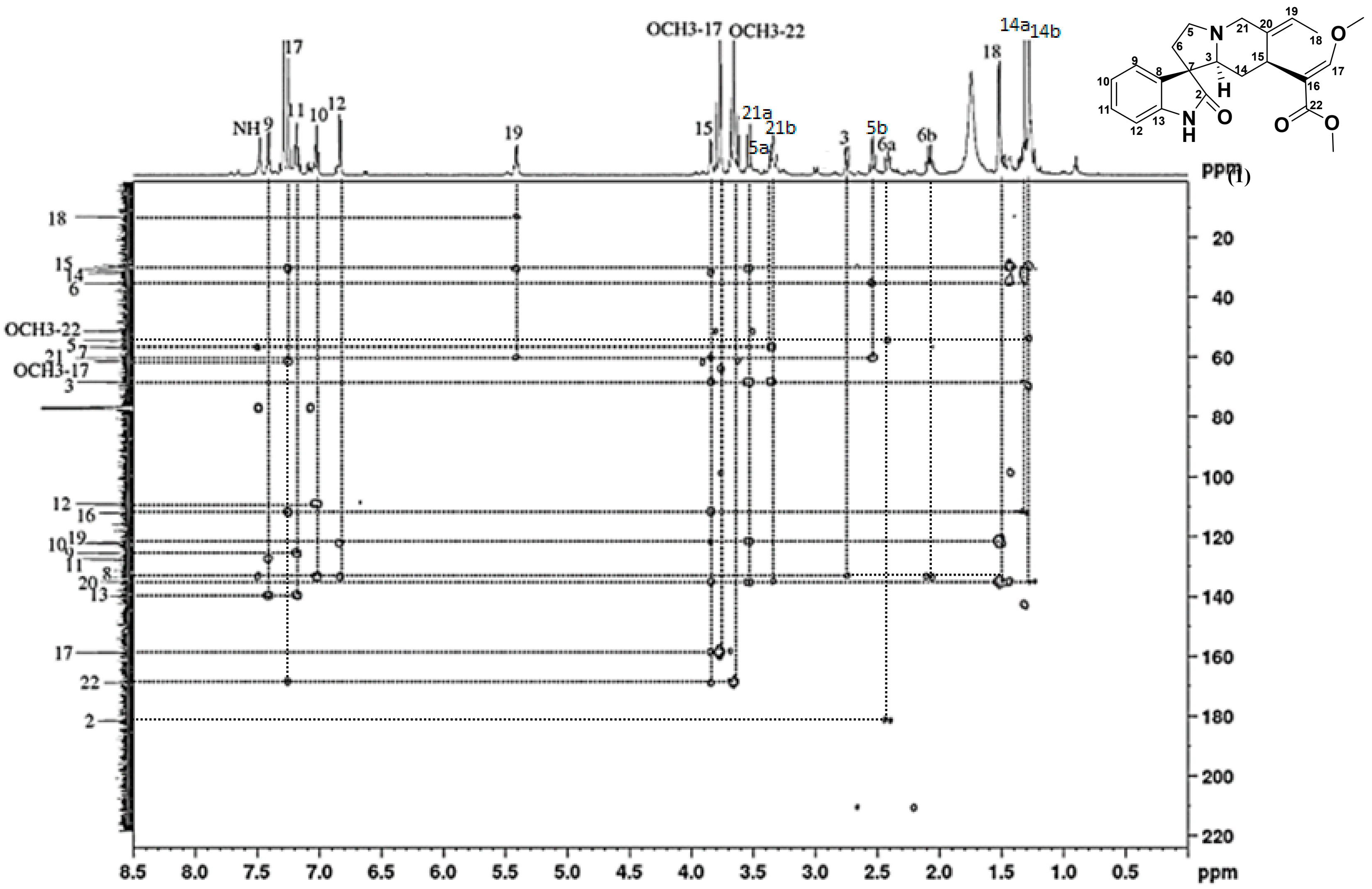

Figure S11: HMBC spectrum of compound (**1**) (CDCl3, 500MHz).

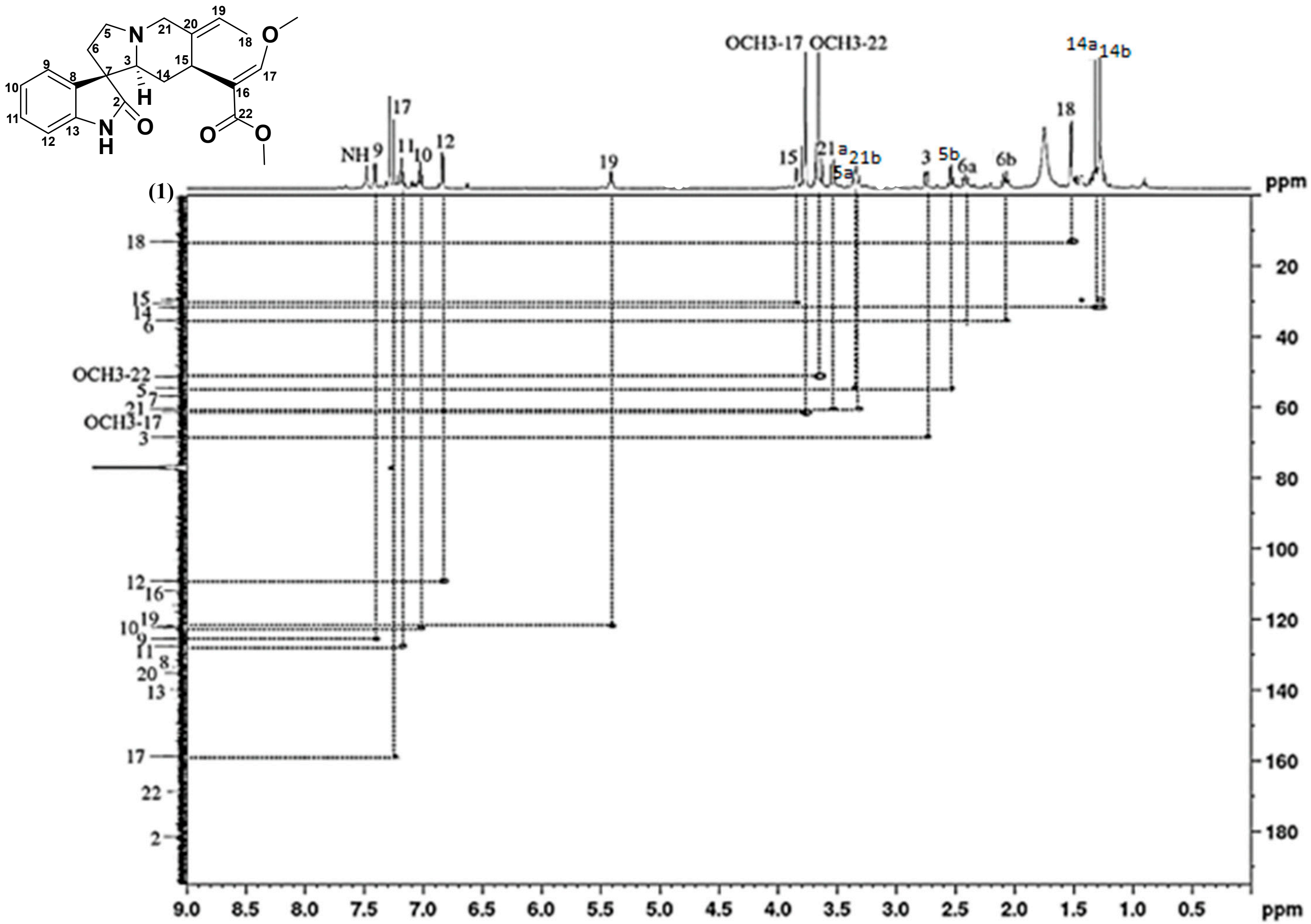

Figure S12: HSQC spectrum of compound (**1**) ($CDCl_3$, 500MHz).

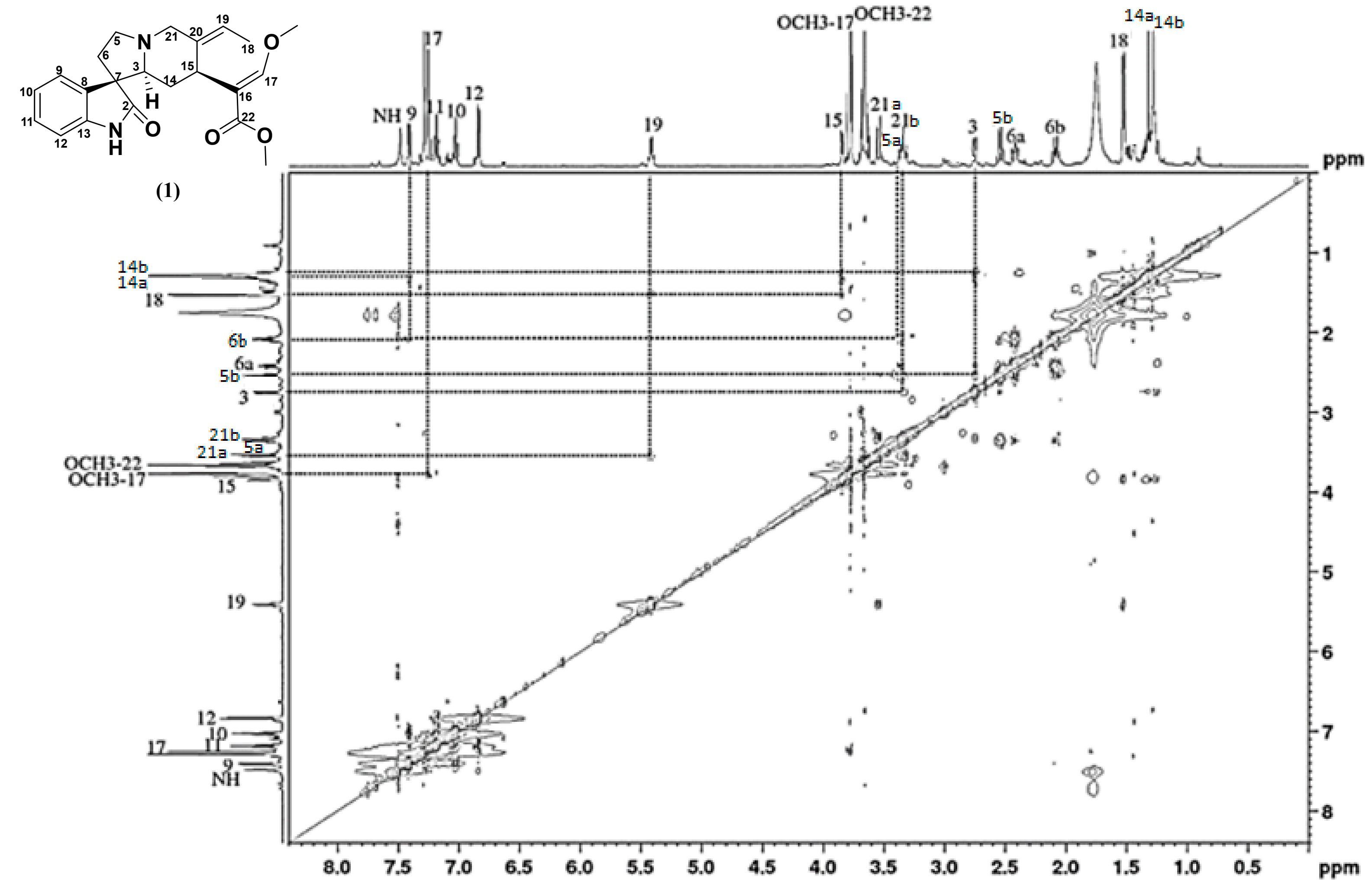

Figure S13: NOESY spectrum of compound (**1**) ($CDCl_3$, 500MHz).

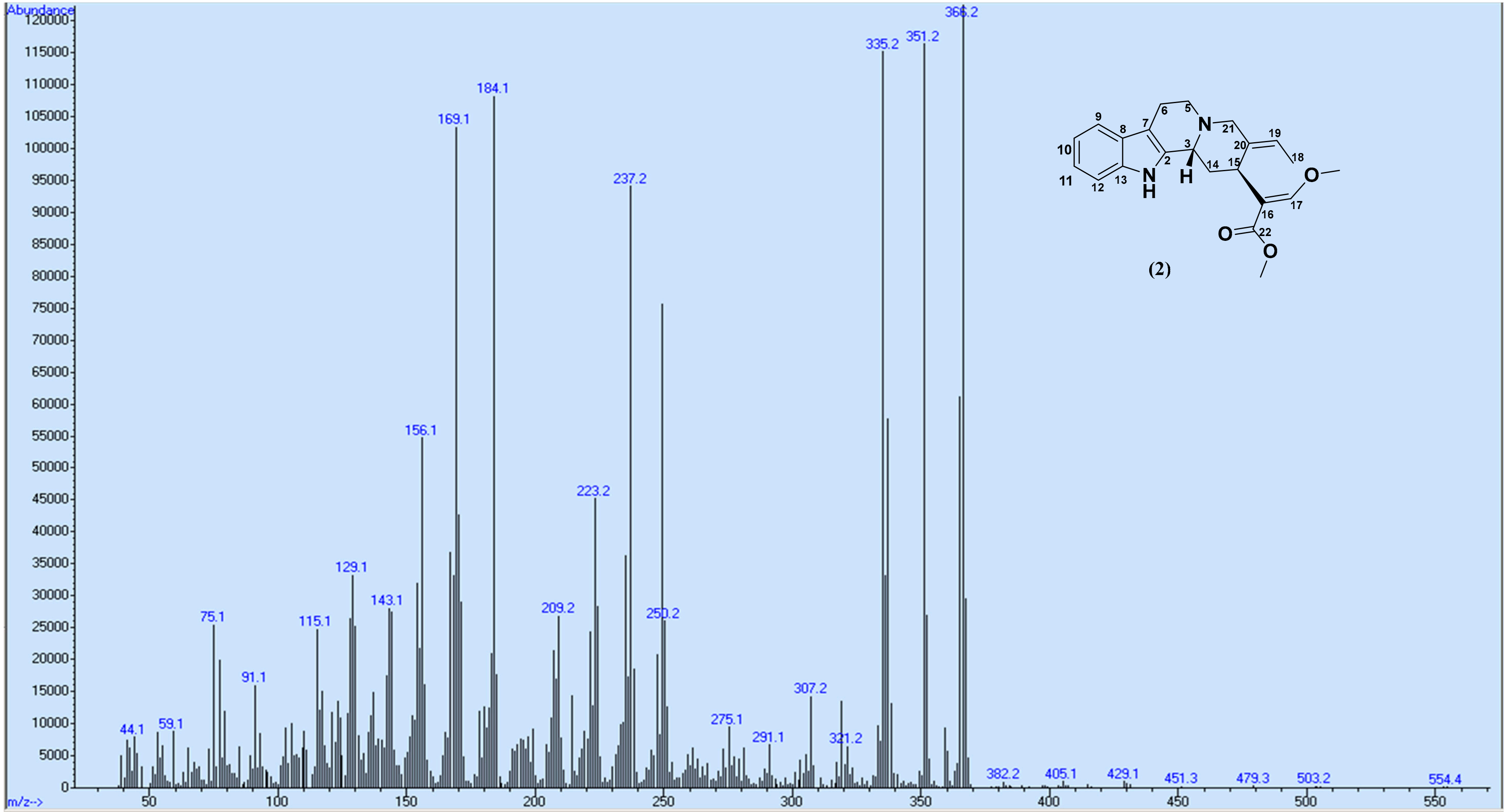

Figure S14: GC-EI-MS spectrum of compound (**2**).

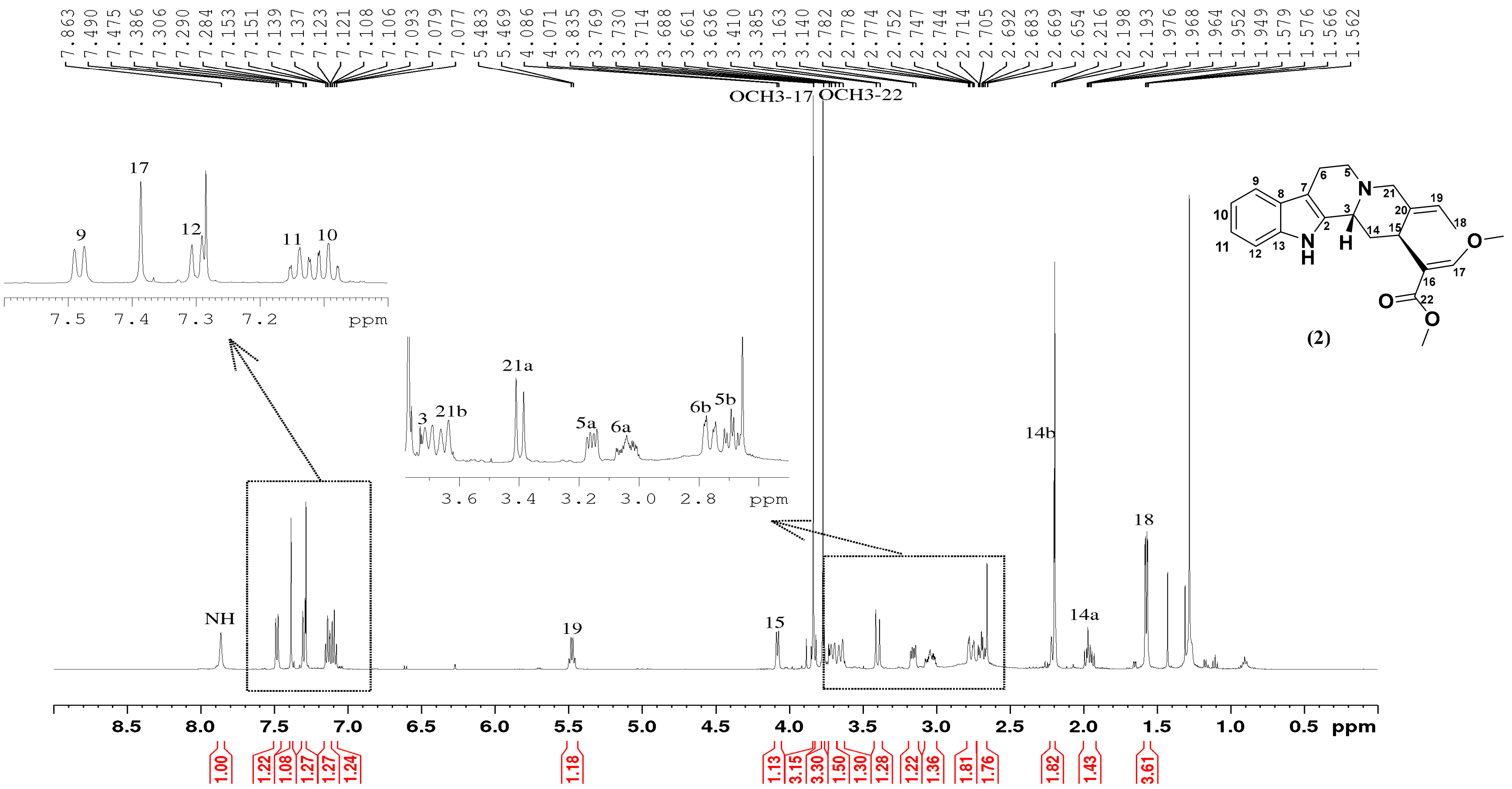

Figure S15: ^{1}H NMR spectrum of compound (**2**) (500 MHz, CDCl$_3$).

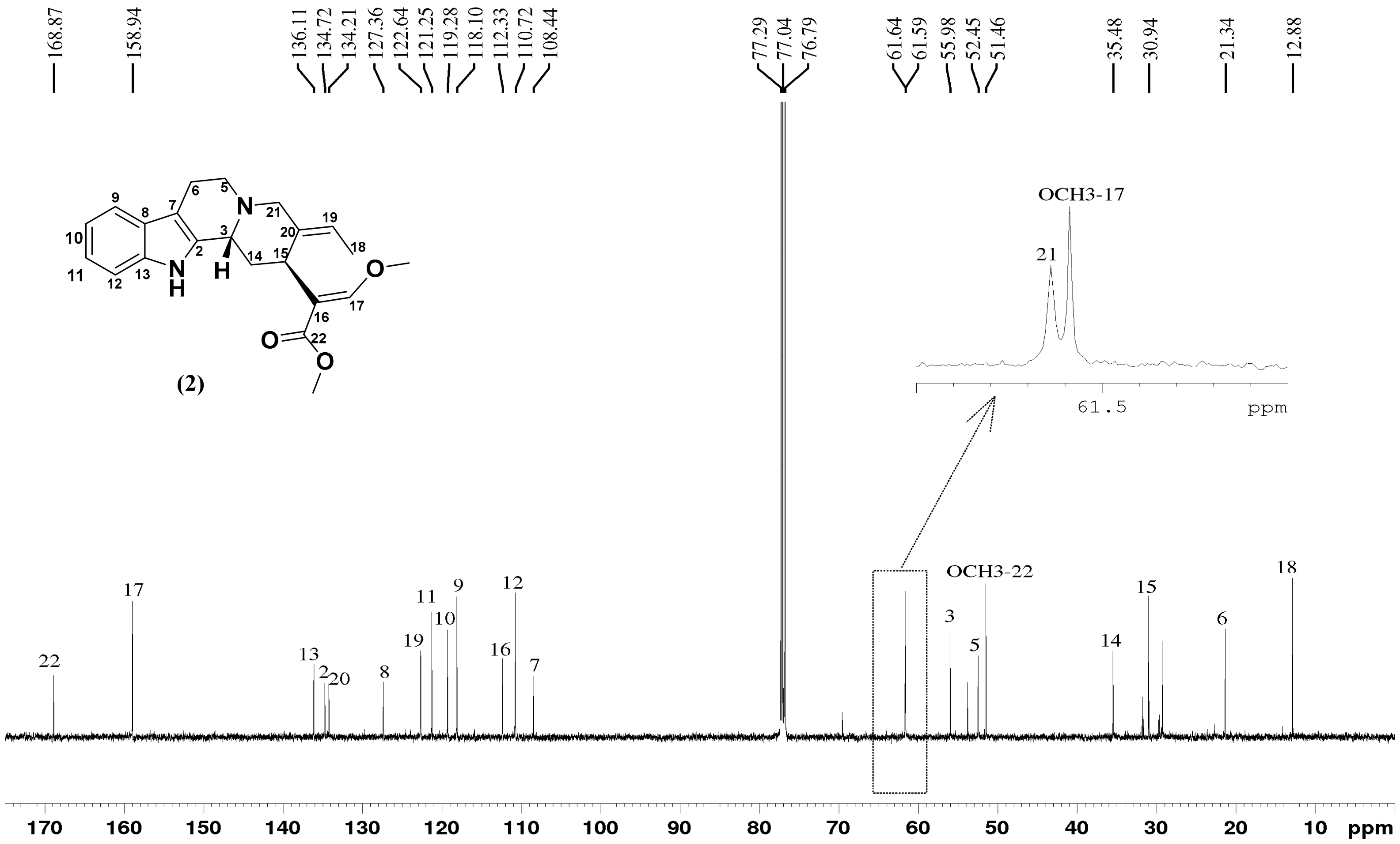

Figure S16: ^{13}C NMR spectrum of compound (**2**) (CDCl$_3$, 125MHz).

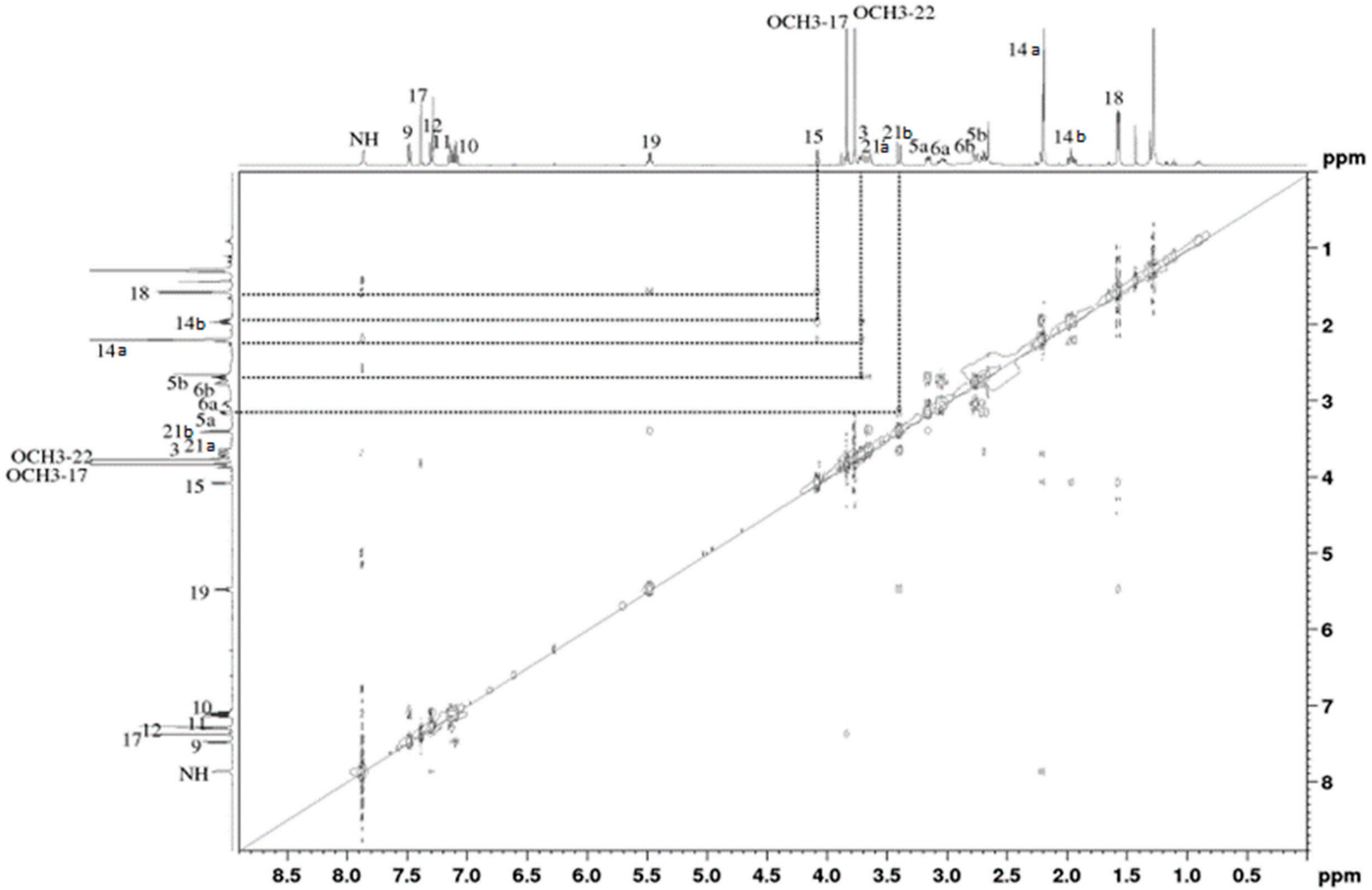

Figure S17: NOESY spectrum of compound (**2**) ($CDCl_3$, 500 MHz).

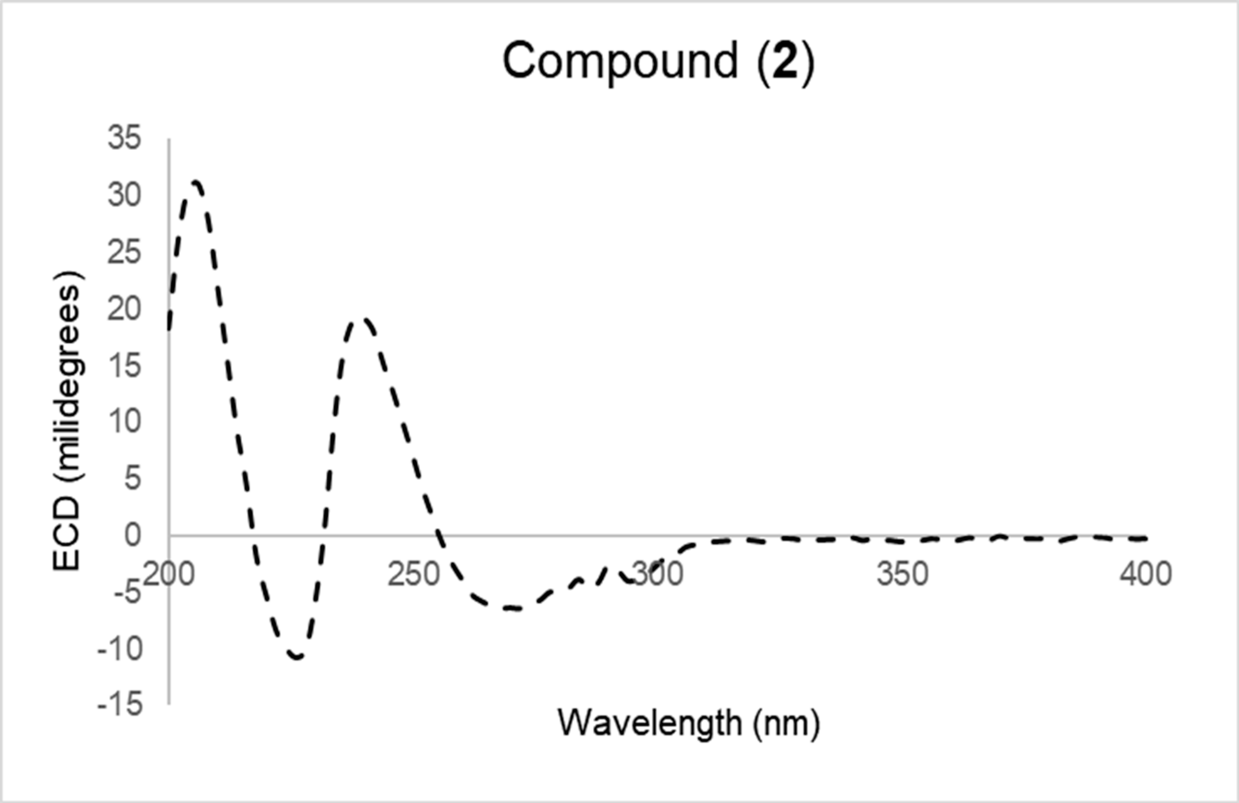

Figure S18: ECD spectrum of compound (**2**) (MeOH, 0.2 mg/mL).

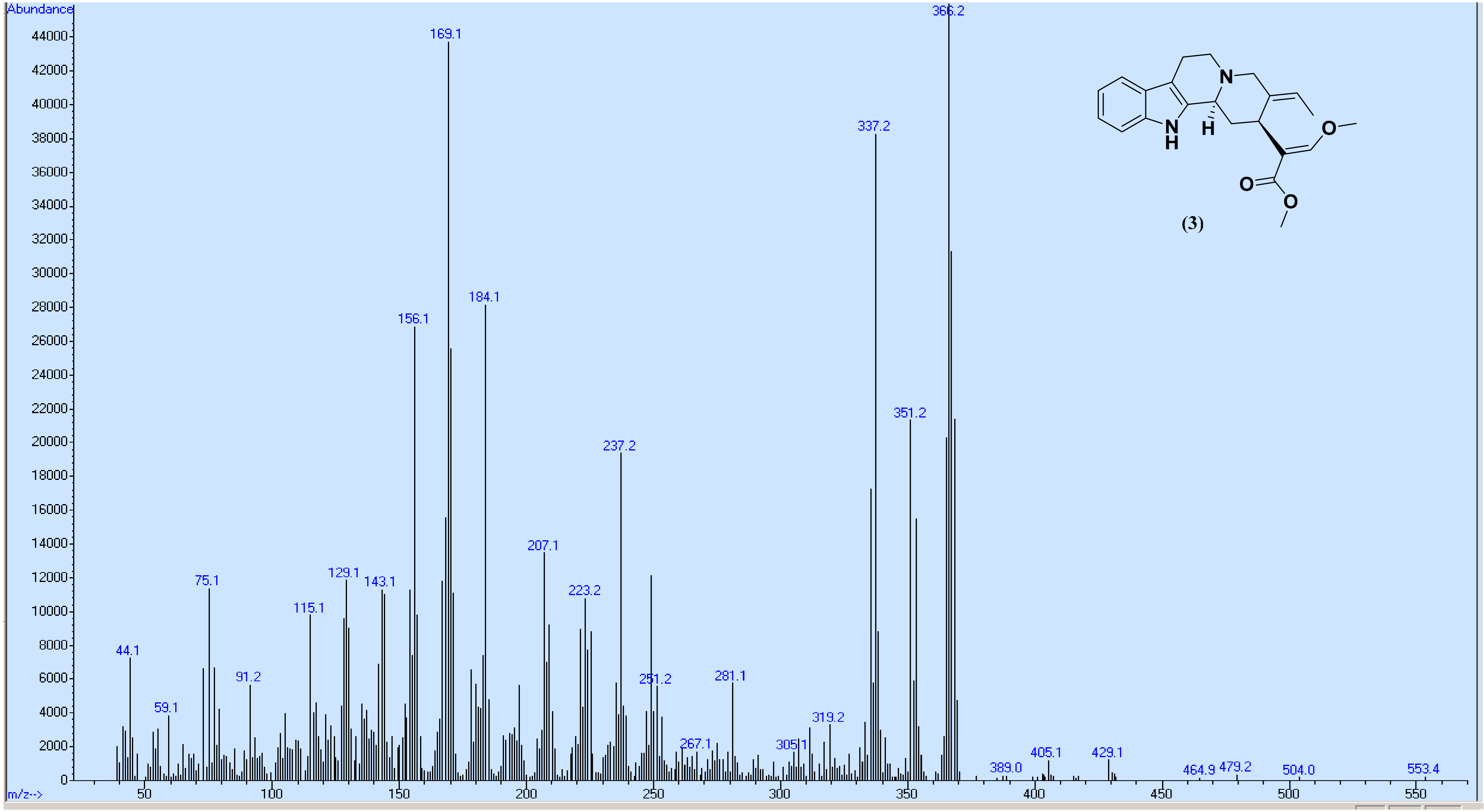

Figure S19: GC-EI-MS spectrum of compound (**3**).

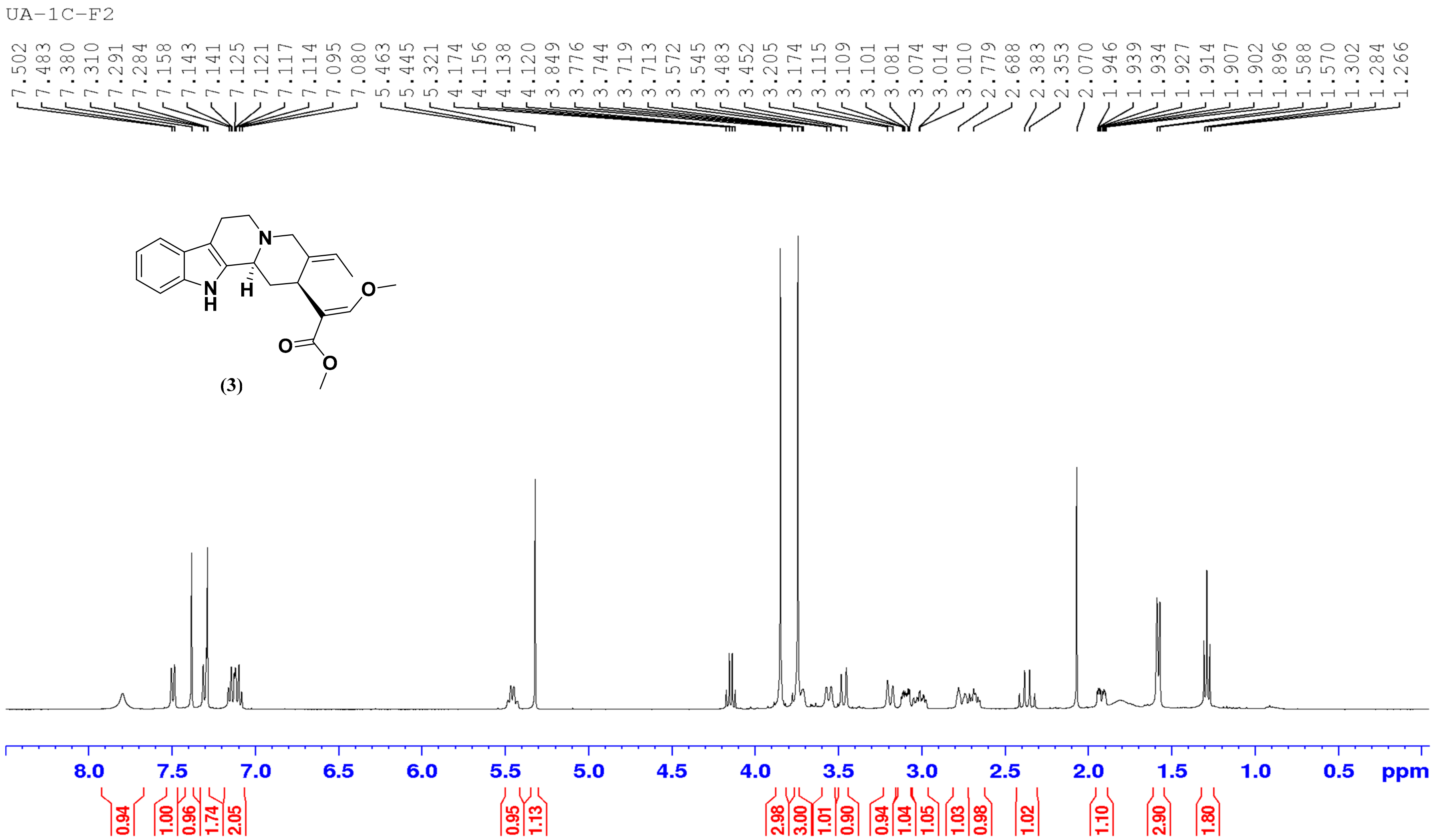

Figure S20: ^{1}H NMR spectrum of compound (**3**) (500 MHz, CDCl3).

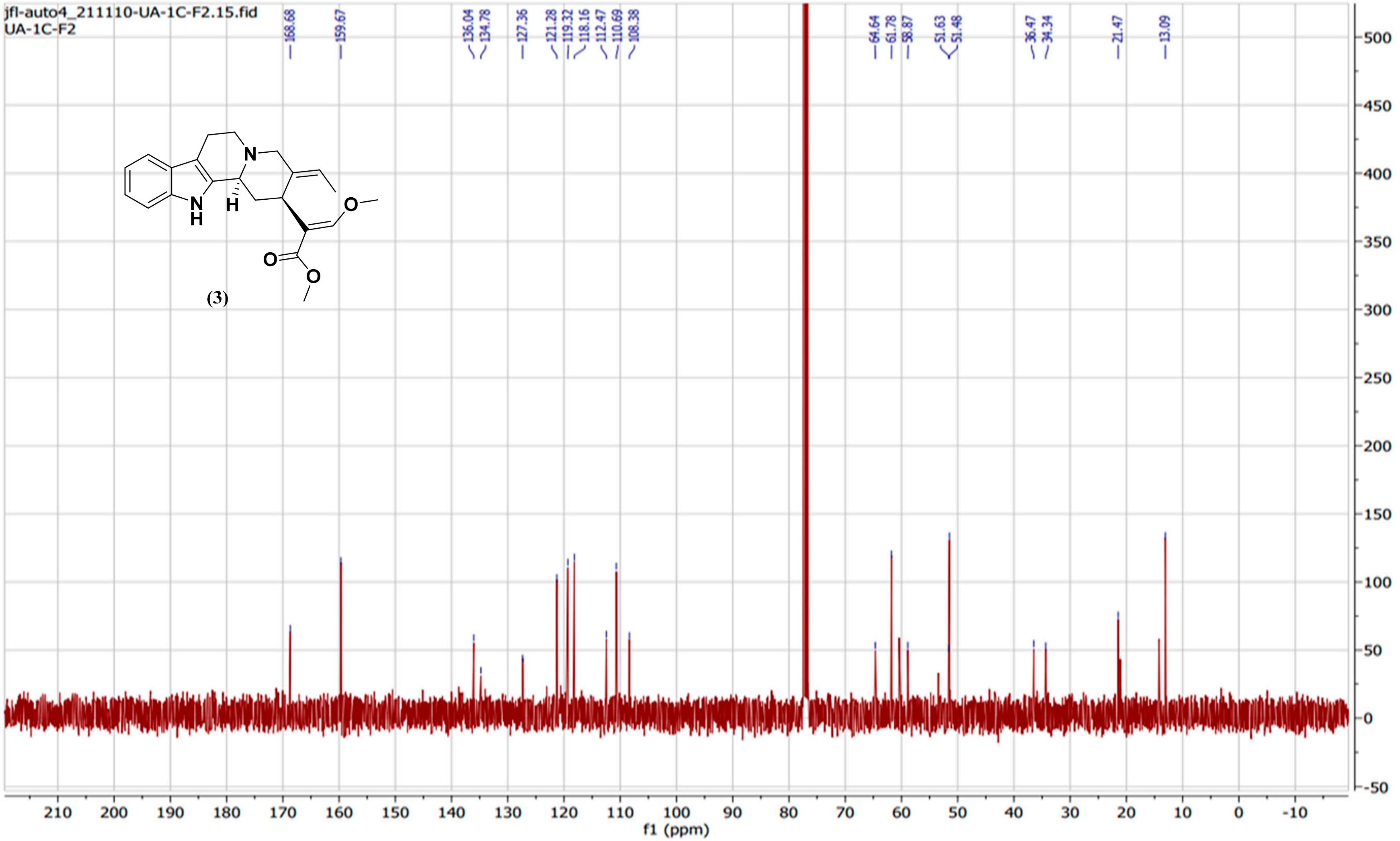

Figure S21: ^{1}H NMR spectrum of compound (**3**) (500 MHz, $CDCl_3$).

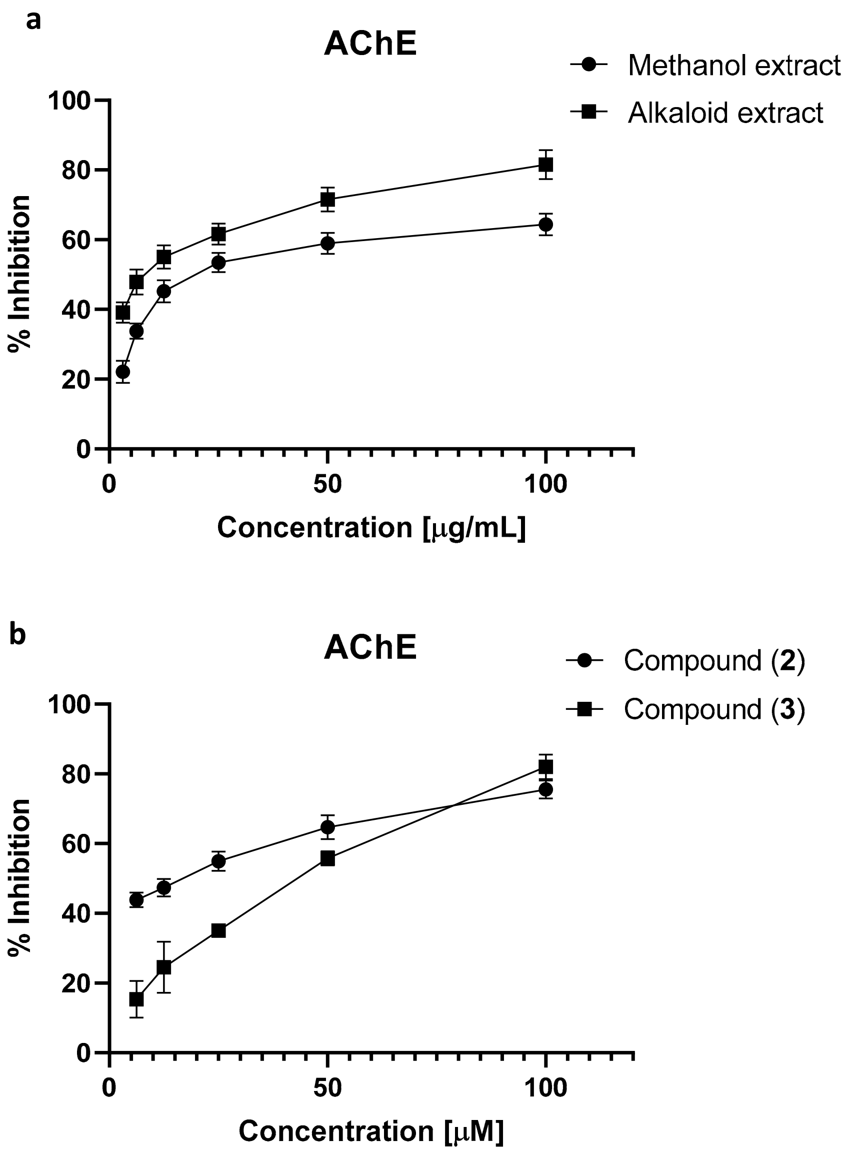

Figure S22: The dose-dependent curve of (a) methanol and alkaloid extracts and (b) compounds (**2**) & (**3**) against acetylcholinesterase (AChE).

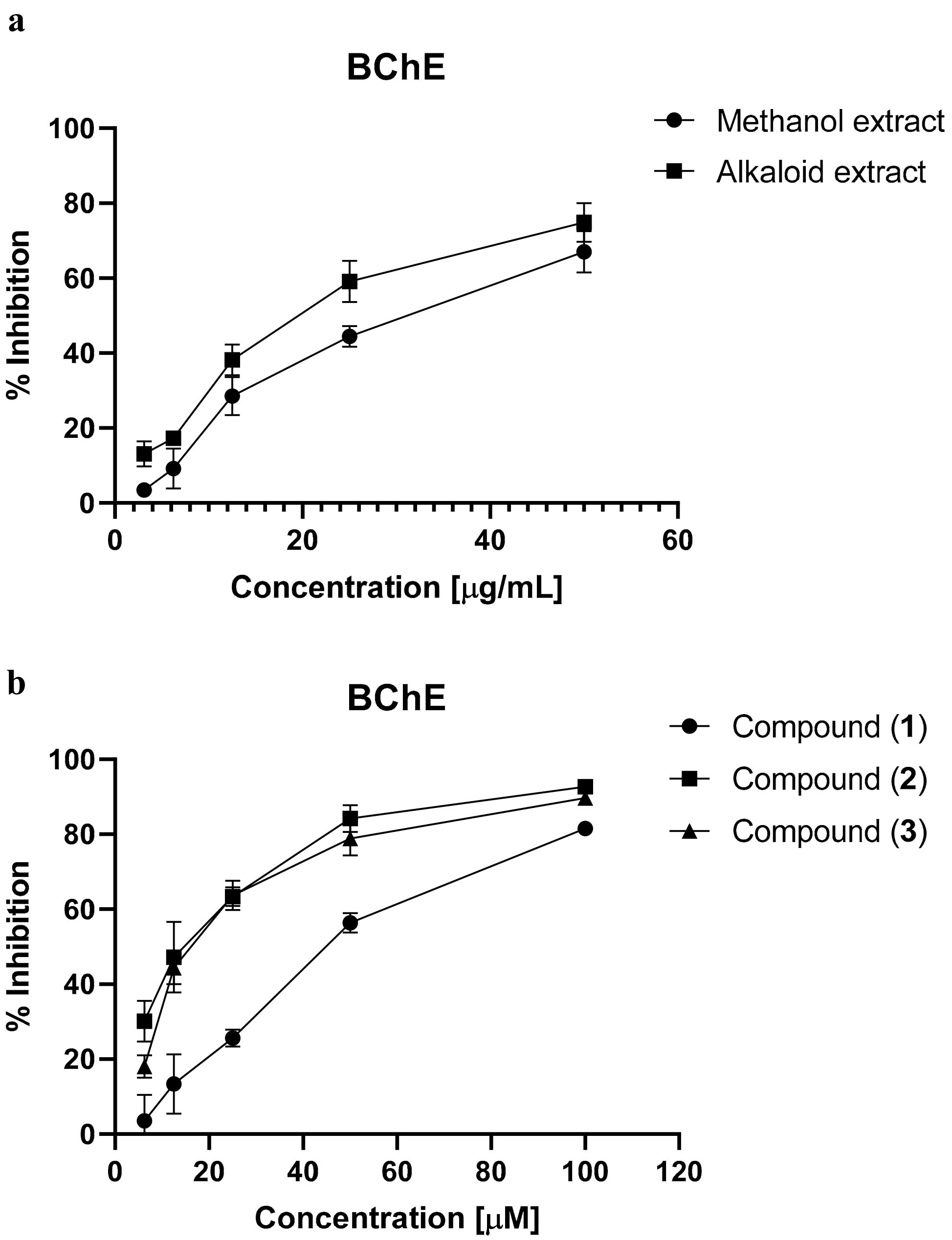

Figure S23: The dose-dependent curve of (a) methanol and alkaloid extracts, and (b) compound 1 – 3 against butyrylcholinesterase (BChE).

Table S1: 1D NMR data of compound (**2**).

	Experimental		Literature (Matsuo et al., 2011)	
**Position**	**δ_H (n, *J*)**	**δ_C, type**	**δ_H (n, *J*)**	**δ_C, type**
NH	7.86 s (1H)	-	7.71 s (1H)	-
2	-	134.7, C	-	134.8, C
3	3.72 br d (1H, 12.1)	56.0, CH	3.69 dd (1H, 12.0, 2.0)	56.0, CH
5a	3.14 dd (1H, 11.1, 4.6)	52.5, CH_2	3.10 dd (1H, 11.0, 5.5)	52.5, CH_2
5b	2.66 ddd (1H, 11.5, 4.3)	52.5, CH_2	2.65 ddd (1H, 11.0, 4.2)	52.5, CH_2
6a	3.04 m (1H)	21.3, CH_2	3.00 m (1H)	21.4, CH_2
6b	2.76 br d (1H, 15.1)	21.3, CH_2	2.73 br d (1H, 15.0)	21.4, CH_2
7	-	108.4, C	-	108.3, C
8	-	127.4, C	-	127.3, C
9	7.48 d (1H, 7.2)	118.1, CH	7.45 d (1H, 7.2)	118, CH
10	7.08 td (1H, 7.2, 1.0)	119.3, CH	7.07 td (1H, 7.2, 1.0)	119.2, CH
11	7.12 td (1H, 7.2, 1.0)	121.3, CH	7.11 td (1H, 7.2, 1.0)	121.1, CH
12	7.29 d (1H, 7.2)	110.7, CH	7.28 d (1H, 7.2)	110.7, CH
13	-	136.1, C	-	136, C
14a	2.19 br d (1H, 13.5)	35.5, CH_2	2.19 br d (1H, 13.4)	35.5, CH_2
14b	1.93 m (1H)	35.5, CH_2	1.90 ddd (1H, 13.4, 12.0, 7.2)	35.5, CH_2
15	4.07 d (1H, 7.0)	30.9, CH	4.05 d (1H, 7.2)	30.9, CH
16	-	112.3, C	-	112.3, C
17	7.39 s (1H)	158.9, CH	7.36 s (1H)	158.8, CH
18	1.56 dd (3H, 6.8, 1.8)	12.9, CH_3	1.55 dd (1H, 6.8, 1.7)	12.8, CH_3
19	5.47 q (1H, 6.7)	122.6, CH	5.45 q (1H, 6.8)	122.5, CH
20	-	134.2, C	-	134.2, C
21a	3.63 br d (1H, 12.8)	61.6, CH_2	3.62 br d (1H, 13.2)	61.7, CH_2
21b	3.39 br d (1H, 12.8)	61.6, CH_2	3.35 br d (1H, 13.2)	61.7, CH_2
22	-	168.9, C	-	168.8, C
OCH_3-22	3.77 s (3H)	51.5, CH_3	3.73 s (3H)	51.4, CH_3
OCH_3-17	3.84 s (3H)	61.6, CH_3	3.82 s (3H)	61.5, CH_3

δ=chemical shift (ppm); *J* = coupling constant (Hz); n = no. of proton

Table S2: 1D NMR data of compound (**3**).

	Experimental		Literature (Takayama et al., 1992) [22]	
**Position**	**δ_H (n, *J*) (400 MHz)**	**δ_C, type (100 MHz)**	**δ_H (n, *J*) (500 MHz)**	**δ_C, type (125 MHz)**
NH	7.80 br s (1H)	-	7.79 br s (1H)	-
2	-	134.8, C	-	134.8, C
3	3.56 br d (1H, 11.2)	58.9, CH	3.52 dd (1H, 11.3, 2.0)	58.8, CH
5a	3.09 m (1H)	51.5, CH_2	3.07 ddd (1H, 11.1, 5.4, 3.2)	51.6, CH_2
5b	2.68 ddd (1H, 11.0, 10.0, 4.7)	51.5, CH_2	2.65 ddd (1H, 11.1, 9.6, 4.6)	51.6, CH_2
6a	3.00 m (1H)	21.5, CH_2	2.98 ddd (1H, 15.0, 9.6, 5.4, 2.0)	21.5, CH_2
6b	2.76 br d (1H, 15.0)	21.5, CH_2	2.73 br d (1H, 15.0)	21.5, CH_2
7	-	108.4, C	-	108.3, C
8	-	127.4, C	-	127.3, C
9	7.49 d (1H, 7.7)	118.2, CH	7.46 dd (1H, 7.8, 1.2)	118.1, CH
10	7.10 td (1H, 7.7, 1.2)	119.3, CH	7.07 td (1H, 7.8, 1.2)	119.3, CH
11	7.14 td (1H, 7.7, 1.2)	121.3, CH	7.11 td (1H, 7.8, 1.2)	121.1, CH
12	7.30 d (1H, 7.7)	110.7, CH	7.26 dd (1H, 7.8, 1.2)	110.7, CH
13	-	136.0, C	-	136.0, C
14a	2.37 q (1H, 12.5)	34.3, CH_2	2.33 ddd (1H, 12.5, 12.5, 11.3)	34.3, CH_2
14b	1.92 dq (1H, 12.6, 2.2)	34.3, CH_2	1.89 ddd (1H, 12.5, 5.0, 2.0)	34.3, CH_2
15	3.73 (1H, overlap)	36.5, CH	3.70 d (1H, 12.5)	36.4, CH
16	-	112.5, C	-	112.5, C
17	7.38 s (1H)	159.7, CH	7.35 s (1H)	159.6, CH
18	1.58 br d (3H, 7.12)	13.1, CH_3	1.55 dt (1H, 7.2, 1.4)	13.1, CH_3
19	5.45 q (1H, 7.2)	120.5, CH	5.42 br q (1H, 7.2)	120.4, CH
20	-	133.7, C	-	134.0, C
21a	3.47 d (1H, 12.5)	64.6, CH_2	3.44 d (1H, 12.5)	64.6, CH_2
21b	3.19 br d (1H, 12.5)	64.6, CH_2	3.16 dd (1H, 12.5, 1.0)	64.6, CH_2
22	-	168.7, C	-	168.7, C
OCH_3-22	3.75 s (3H)	51.6, CH_3	3.72 s (3H)	51.4, CH_3
OCH_3-17	3.85 s (3H)	61.8, CH_3	3.82 s (3H)	61.7, CH_3

δ= chemical shift (ppm); *J* = coupling constant (Hz); n = no. of proton
